# “It shows that I belong”: a qualitative study assessing birth companionship of choice in a maternity clinic in Freetown, Sierra Leone

**DOI:** 10.1186/s12884-025-07711-z

**Published:** 2025-05-27

**Authors:** Olivia Munson, Joan H. Shepherd, Abubakarr Lewally, Foday Musa Janneh, Stefanie Theuring

**Affiliations:** 1 Charité – University Medicine, Augustenburger Platz 1, 13353 Berlin, Germany; 2 National School of Midwifery, Fourah Bay Road, Freetown, Sierra Leone; 3 Princess Christian Maternity Hospital, Fourah Bay Road, Freetown, Sierra Leone

**Keywords:** Sierra Leone, Birth companionship of choice, Respectful maternity care, Woman-centered care

## Abstract

**Background:**

Despite existing evidence supporting birth companionship of choice as a simple, low-cost measure to improve quality of care in childbirth, it is inconsistently practiced in many regions. In Sierra Leone, one of the least safe countries for women to deliver a baby, Princess Christian Maternity Hospital (PCMH) began encouraging companionship of choice for women during childbirth in March 2022. However, little is known about the perspectives of healthcare workers or the perinatal experiences of women and their companions. Our objective was to describe the experiences and perceptions surrounding birth companionship of choice among healthcare workers, women, and their companions.

**Methods:**

We conducted a qualitative assessment at PCMH in August 2022 through eleven focus group discussions and thirteen in-depth interviews with healthcare workers, women, and their companions. Discussions and interviews were recorded, transcribed, and thematically coded for analysis.

**Results:**

Women described various means of support from their birth companion and cited trust and the ability to provide expertise as key criteria when choosing a companion. Healthcare workers emphasized that, despite initial hesitation, companionship has been advantageous. However, there were still many challenges for healthcare workers when companions were present in the labor ward, such as maintaining patient privacy. Companions revealed that they were learning about facility-based delivery through companionship and sharing their positive experiences with their community. With a few exceptions, both companions and women reported overall satisfaction with their treatment in the labor ward.

**Conclusion:**

Findings suggest that birth companionship of choice is a key component of a positive birth experience, improving perceived quality of care and potentially increasing the number of women giving birth in facilities. It also presents many advantages and challenges for healthcare workers. The presence of a birth companion inside the labor ward increases appreciation for facility-based care and potentially alleviates mistrust between the healthcare system and the community.

**Supplementary Information:**

The online version contains supplementary material available at 10.1186/s12884-025-07711-z.

## Background

While most maternal deaths are preventable, the maternal mortality rate (MMR) remains unacceptably high in many low-resource settings [1]. Efforts to reduce maternal mortality have centered around increasing the proportion of facility-based deliveries (FBDs) [2]. Notable barriers to FBD include institutional obstetric violence and negative perceived quality of care [3, 4].

One strategy that has been shown to increase the number of women choosing FBD as well as women’s satisfaction with healthcare services is encouraging birth companionship of choice (BCC) during childbirth [4], defined from early labor through at least the birth [5]. Part of the WHO Respectful Maternity Care recommendation [6], continuous one-to-one support by a trusted person of choice provides numerous benefits for both mothers and newborns and is considered a universal human right in the context of the right to respectful maternity care [7]. A 2017 Cochrane review [5] demonstrated that women who received continuous one-to-one support were more likely to have spontaneous vaginal birth and shorter labors and less likely to require analgesia. Similar findings have been reported in Brazil and Nepal [8, 9], where lack of companionship was shown to increase the chance of caesarean delivery. BCC may also lead to improved outcomes for newborns and is associated with an increased five-minute APGAR score [10]. Women who are accompanied during childbirth are less likely to experience mistreatment [11–13] and more likely to be satisfied with their received care [14, 15]. By ensuring women’s autonomy and agency during childbirth, engaging communities and health workers in the implementation of sustainable and respectful labor practices, and improving health systems to respect women’s sexual and reproductive rights including the right to privacy and confidentiality, BCC is a critical strategy to improve maternal and newborn care [16].

Despite the numerous benefits of this simple, low-cost measure, BCC is not consistently practiced or even tolerated, particularly in low- and middle-income countries [6, 17, 18]. Studies in Kenya and Ethiopia [19–21] revealed that less than 30% of mothers had a companion during childbirth. Barriers cited by health providers include space restrictions, hygiene concerns, wariness of interference with clinical decisions, and a lack of awareness of the benefits associated with BCC [17, 22–24]. However, studies in Malawi, Tanzania, and Nigeria [15, 25, 26] have shown that BCC is well received by healthcare workers (HCWs), with many expressing that they would want the initiative to continue.

In Sierra Leone, MMR has decreased tremendously over the last decades, from 1682 deaths per 100,000 live births in 2000 to 443 in 2020 [1], due to efforts such as establishing a peripheral health unit network [23] and providing free healthcare coverage for pregnant women, breastfeeding mothers, and infants [27]. However, there is still a 1 in 52-lifetime risk of dying due to pregnancy or childbirth [1], and mistrust in the existing health system prevails, especially since the Ebola outbreak of 2014–2016 [27]. As part of its efforts to improve maternal quality of care and reduce the MMR, the Ministry of Health and Sanitation, through collaborative partnership with some institutions such as the Charité - Universitätsmedizin in Berlin, began promoting respectful maternity care [28]. Previous research conducted at PCMH has demonstrated a lack of understanding and acceptance among HCWs towards BCC as well as barriers to implementation of BCC such as lack of space [23]. In March 2022, the national referral maternity hospital in Freetown, Princess Christian Maternity Hospital (PCMH), started a campaign to raise awareness about the benefits of BCC among HCWs and encourage the implementation of BCC in its facilities.

In a setting where healthcare providers have traditionally been resistant to the presence of companions and where there is a lack of trust between patients and the healthcare system, successfully implementing BCC requires a thorough understanding of perspectives regarding companionship. Our objective was to provide actionable insights about the experiences and perceptions surrounding BCC among delivering women, HCWs, and birth companions at PCMH. Through an in-depth exploration, the study intends to shed light on the intricacies involved in integrating BCC in such a complex setting, thereby facilitating the effective implementation of BCC and improving quality of maternal care.

## Methods

We conducted a qualitative assessment in August 2022 using Focus Group Discussions (FGDs) and individual in-depth interviews (IDIs) to evaluate BCC at PCMH. The qualitative research design was chosen because, by privileging participant voices, it provides space for participants to express their personal views and experiences in depth and offers the opportunity for new insights.

### Setting

PCMH is a teaching-tertiary referral hospital, providing comprehensive emergency obstetric services throughout the Western Area province, and ultimately for the entire country [29]. Similar to the other provinces, Western Area has underdeveloped infrastructure, with limited access to clean water, sanitation facilities, and reliable electricity. Poorly maintained roads and a lack of efficient public transportation hinder mobility and access to healthcare services, especially during the rainy season [27]. High levels of poverty affect much of the population, and the under-resourced healthcare system faces shortages of personnel, equipment, and supplies.

Beginning March 2022, PCMH systematically promoted BCC through staff training and restructuring of the labor ward to increase privacy spaces. This initiative was supported by the Ministry of Health and Sanitation and the WHO Sierra Leone, who developed a Respectful Maternity Care Training Manual [30]. PCMH staff took part in a three-day workshop in March 2022 emphasizing the critical role of BCC in improving health outcomes and addressing gaps in respectful maternity care by enhancing the woman’s sense of autonomy and comfort. The training aimed at positively changing HCWs’ attitudes towards birth companions. Group discussions and activities were incorporated to help participants reflect on the practical implications of implementing BCC and consider solutions to foreseen challenges.

### Participant eligibility & recruitment

The target population of our study was individuals experiencing BCC at PCMH between March and August 2022. This population can be divided into three distinct eligible groups: (1) women who delivered with a birth companion, (2) nurses and midwives (referred to here as HCWs) attending to deliveries with BCC, and (3) persons who accompanied a delivering woman. Eligibility for group 1 required postpartum health condition allowing for interview; for all three groups, it required informed consent. No underage participants were included in the study.

Eligible participants were purposively identified and approached by staff at PCMH for all three groups. Women and birth companions were called by PCMH staff via telephone and invited to participate in the study upon clarifying eligibility. Participation among HCWs was determined by the head nurse, who identified eligible and willing HCWs in accordance with staff requirements of the labor ward.

### Implementation of FGDs and IDIs

Data was collected by a trained study staff member through FGDs and IDIs. The FGDs and IDIs were semi-structured, using prepared open-ended questions to lead but not limit the discussion. Follow-up questions and probing were used to gather details and depth about participants’ experiences.

FGDs consisted of two to four participants. The size and composition of FGDs within the three distinct groups were determined according to time availability at the facility. FGDs and IDIs were conducted in a private room within PMCH and led by two researchers, one male local staff and the other a female researcher from a German institution with no previous ties to Sierra Leone. Neither researcher had ties to participants and both engaged in critical reflection on potential bias and their positions toward the participants. Researchers introduced themselves to participants and informed the participants about the goals and the content of the study. Participants could choose to speak in English or Krio, with Krio interpretation provided by local staff. Discussions and interviews lasted 20 to 90 min according to the willingness of the participants. All interviews and discussions were recorded; additionally, study staff took notes manually. No repeat interviews were carried out. The total number of FGDs and IDIs reflects achievement of data saturation, meaning that interviews and discussions did not yield any new information.

### Data analysis

The recorded discussions and interviews were manually transcribed *verbatim*, using notes to clarify contextual meanings. In order to maintain the immediacy of the discussions, we chose not to return transcripts to participants for comment. We performed a data-driven, thematic analysis by inductively coding the raw data to identify existing units and patterns. Subsequently, codes were synthesized to compare and organize recurring patterns and to determine relationships between them. Major categories were identified, then grouped and summarized into several key themes and sub-themes. The socio-ecological model was used as a theoretical framework for understanding the intricate interactions between individual, interpersonal, institutional, and community factors that influence the successful implementation of BCC and respectful maternity care [31]. Data was initially coded using an open-source qualitative coding and analysis program, TAMS Analyzer [32], and then printed and organized manually for the synthesizing process. Researchers reached a consensus on all final coded data and checked coder reliability using criteria outlined by the Standards for Reporting Qualitative Research [33].

## Results

In total, 45 individuals participated in our study, representing fifteen individuals from each of the three groups. No participants refused to participate or continue with the study. Data collection comprised a total of eleven FGDs (32 participants) and thirteen IDIs; in group 1, four FGDs and six IDIs; in group 2, three FGDs and three IDIs; and in group 3, four FGDs and four IDIs. Individuals in the participant group of delivering women were 19 to 32 years old with an average of 26 years (*n* = 14, 1 missing data). In the group of HCWs, ages ranged from 26 to 58 years with an average of 41 years (*n* = 15), and participants in the group of birth companions were 23 to 56 years old with an average of 35 years (*n* = 14, 1 missing data). Four out of the fifteen companions were male. Of the women delivering, twelve were married, one engaged, and two single. Five women had given birth to their first child. All participants were residents of Freetown.

The categories, themes and sub-themes identified in the FGDs and IDIs as well as their corresponding socio-ecological levels are presented in Table 1. Additional evidence supporting the identified themes and sub-themes can be found in a supplementary file [see Additional file 1].

**Table 1:** Description of identified categories, themes, and sub-themes

Categories	Socio-ecological Level	Themes	Sub-Themes
Types of support provided by companions´	Individual/Interpersonal	Practical support	Gathering supplies
			Hygiene
			Food and water
			Assistance with baby
		Informational support	Medical reminders
			Advice and guidance
			Communicate with relatives outside
		Advocacy	Companion can call for help
			Protection from abuse
		Comfort	Physical touch
			Prayer
			Physical comfort
			Familiarity
		Emotional support	Encouragement and strength
			Companion as proxy
Choosing a companion	Individual/Interpersonal	Male vs. female companions	Better treatment from husbands after witnessing birth
			Empathy and emotional support
			Increased bonding, unites the family
			Men are afraid
			Women provide more expertise
		Trust	Fear of witchcraft
			Trust by giving good advice
			Trust to be non-judgmental and confidential
			Trust by sharing everything
			Trust by support during pregnancy
Implementing companionship	Institutional	Reasons for previously not allowing companionship	Concerns about privacy
			Threat of theft or scamming
			Lack of space and resources
			Arguments with relatives
		Educating the companion	
		Monitoring the companion	
		Knowledge of importance of companionship	Seniors were not implementing
			Lack of knowledge
		Workshop, institutionalization means easier implementation	
		Satisfaction with companionship	
Advantages for HCWs	Individual/Institutional	Reduced workload	Companions help physically during labor
			Companions can fetch medical supplies
			Relief from providing emotional support
		Communication	Language barriers and translation
			Companions provide reliable information
		Companion as witness	Protect nurse from false claims of abuse
			Protect nurse from false claims of baby theft
		Improve patient cooperation	Companions know the patient best
			Patients become more cooperative
Disadvantages for HCWs	Individual/Institutional	Inappropriate behavior	Encourage or engage in physical and emotional abuse
			Companions may make the patient less cooperative
		Interference	Interference with medical processes
			Native herbs
			Dangerous encouragements
		Privacy	HIV and stigma
			Companions curious about other patients
			Companions talk outside about what they witnessed
		Extra work	Extra materials necessary
			Companions faint and require attention
Companionship and the community	Community	Home vs. hospital delivery	
		Companion is learning about FBDs	
		Treatment from HCWs	Satisfaction with treatment
			Mistreatment and abuse
		Sharing about experience with community	Companions give accurate information to community
			Encourage FBD in community

### Introducing BCC to PCMH

Before staff training workshops, HCWs reported a lack of knowledge about the importance of companionship. While the idea was familiar to some, it was not thought to be relevant or appreciated.

*When we started doing delivery*,* that’s what we met our seniors doing. They don’t allow relatives to be in the labor ward.* Healthcare Worker, FGD5.

*When we go to our midwifery schools*,* the idea was there*,* but like in our setting we don’t know if the people really appreciate that or needed that.* Healthcare Worker, FGD1.

Reasons for an initial hesitation towards companionship included concern about a lack of privacy, space, or resources, such as extra scrubs in the operating theatre. HCWs also reported apprehension that companions might cause trouble, including false accusations, particularly when patients arrived with multiple relatives expecting to attend the birth. Furthermore, HCWs were uncertain about the identity of companions and suspicious of theft or scamming.

*For some of them birth companion*,* they do not come with one*,* they come with three*,* four relatives. So some of them*,* they quarrel (…) They say all sorts of talk against us.* Healthcare Worker, FGD5.

*Sometimes*,* these people that are coming are not the relative of the particular clients (…) We have seen cases*,* (…) this lady was talking to the client admitted to the ward 6. We thought she was a relative to the patient. She steal the patients’ phones!* Healthcare Worker, FGD2.

Despite initial resistance, HCWs reported being highly satisfied with the newly introduced BCC. They also emphasized that institutionalized companionship through formal staff training increased acceptance for the change and made companionship easier to implement with fellow staff members.

*The good outweighs the bad. Maybe there is a hundred good*,* there is just one bad.* Healthcare Worker, FGD1.

### Choosing a companion

When choosing a companion, delivering women and companions both cited trust as a key factor. A woman might trust a companion for reliably being there during the pregnancy or offering her sound advice, or because the companion would be able to keep secrets regarding what they witnessed during the birth.

*Whatever advice she render*,* when I follow that advice*,* it will come out positive. Yes*,* so I trust her.* Delivering Woman, IDI6, age 29.

*If my mother-in-law is there*,* I’m not worried about privacy. But if it had been my sister*,* I’m worried about privacy issue… anytime we will be having disagreement*,* or there will be quarrel with my sister*,* my sister would (…) rub it on my face that*,* the time you were giving birth you were crying like a baby*,* you were helpless*,* I was the one doing x y and z for you and your baby. So I wouldn’t want my sister to tell me all of these things.* Delivering Woman, IDI5, age 19.

Often participants cited concern that someone who is not trustworthy might employ witchcraft to complicate the delivery. This concern was so pronounced that several participants came to the hospital in secret.

*At times*,* when somebody is pregnant*,* for example we are going to the hospital*,* then you started telling everybody*,* I’m going to the hospital! (…) You don’t know*,* because there are so many evil people out there that don’t want you to give birth… They have their own ceremony that they will process for you when you came into the labor room*,* it will become difficult.* Delivering Woman, FGD10.

Most participants expressed that an experienced woman would make the best companion, as she would be able to provide guidance throughout the birthing process, be more understanding, and be of greater assistance in taking care of the baby. Some worried that a husband would care more about the well-being of the baby than the mother. Overall, men were thought to be ineffective companions as they might be too afraid to provide adequate support.

*Some husband*,* they only need the baby… After they have seen the baby*,* they wouldn’t want to know what’s the status of the mother*,* because it’s the baby they’re there for. But my sister*,* my sister can be there for all of us.* Delivering Woman, IDI12, age unknown.

*I do not have the gut to stay and watch my wife put to bed. No*,* I am afraid. I do not like seeing the blood and I do not like seeing her crying*,* so I prefer to stay out.* Male Companion, FGD9.

*The husband reassure her*,* support her*,* but the husband*,* they won’t be there for long to see their wife screaming.* Healthcare Worker, FGD5.

In contrast, other participants posited that husbands could offer more emotional comfort than women because they are more impressed by birth. Many participants believed that after witnessing a woman giving birth, a man would have heightened appreciation for her and be less likely to mistreat the woman later.

*For example*,* I say like Sierra Leone*,* some men*,* they do not have respect for women. So when my husband is going to be there*,* he will know that women go through pain to give birth. For that he will have respect. For that he will not abuse you.* Female Companion, IDI7, age 31.

### Advantages of companionship

Companions provided instrumental support to the delivering women by fetching food and water, caring for necessities such as soap and lappas (wrapper garments), changing linens, and assisting with the baby. Additionally, companions could collect medical supplies such as gloves, sutures, and medications, as necessary.

HCWs expressed that BCC lessened their workload and stress levels. As companions took care of patients’ emotional and practical needs, HCWs had more time for their own duties. Some HCWs also expressed satisfaction that companions could physically prevent uncooperative patients from closing their legs.

*By closing the legs*,* it will put [pressure] on the baby*,* and it will suffocate the baby. So the relative*,* the companion will help us… Hold their legs*,* hold their arms.* Healthcare Worker, FGD2.

Companions were also a bridge of communication between patients and HCWs, even translating for patients if necessary. As advocates, companions helped women make their needs known to medical staff and could quickly call for help if a patient required urgent medical attention. If a woman was unable to communicate, companions could also provide the HCWs accurate medical information about the patient.

*Maybe the nurse (…) is supervising two patients*,* and you are not with that patient… The relative is inside. And that patient is having*,* let me say*,* fitting. He or she will call you quickly.* Healthcare Worker, IDI8, age 50.

*Some will travel with bikes and because of the road network*,* some patients will have a rupture*,* they will come to this hospital*,* they will not explain to us exactly what went wrong. But with some relatives*,* they say*,* no*,* nurse*,* this and this happened. She was travelling with a bike so she started bleeding… It really helps us with some cases. They will explain exactly.* Healthcare Worker, IDI8, age 50.

By giving advice and guidance to the women, companions provided key informational support. They could also help ensure that patients comply with medical instructions, such as fasting before an operation or changing wound dressings.

*If the wife forgets to do the sit-bath*,* the husband will remember… So it’s really nice*,* because if you’re alone*,* you will just skip something and say*,* I will do it later. But if you have someone asking you*,* have you done this? Then you will say OK*,* I should do it now.* Healthcare Worker, FGD1.

Several delivering women expressed relief that someone was with them to take responsibility of their case or serve as a link to the healthcare system in case of emergency or death.

*If you die*,* during that process*,* the nurses*,* the doctors*,* it will be difficult for them to reach your people… If your companion is there*,* they will ask that person to sign on your behalf. But if there is no companion*,* so who will sign? If that person dies*,* who will take the responsibility?* Delivering Woman, IDI6, age 29.

The presence of companions calmed participants by ensuring that someone was observing what was happening, potentially even protecting patients from abuse. HCWs also felt at ease by the presence of companions because they could act as witnesses against fabricated abuse allegations.

*If you have a companion they treat you differently*,* they treat you good. Because they know that the companion will – the companion will talk to them*,* like my aunt… So they treat you well*,* because you have someone in there.* Delivering Woman, FGD6.

*When the birth companion is with the patient*,* she can see everything that is happening in the room*,* so*,* there will be no allegation against the nurse. So the nurse will be free to do her work and relax.* Healthcare Worker, FGD1.

Many participants referred to a history of mistrust surrounding PCMH, in which the public believed that medical staff were stealing or exchanging babies. HCWs expressed relief that companions were there to witness the birth and verify the sex and number of newborns, protecting them from wrongful legal action.

*For our safety*,* that is most important. Because now for these law issues here in Sierra Leone… The patients who say*,* when they went to the ultrasound scan*,* I had a girl*,* but now*,* it’s a boy. They have changed my baby. If the birth companion is not there*,* what will we do? So it is very important for our own safety.* Healthcare Worker, FGD1.

Physical touch and prayer were key sources of comfort and courage for delivering women. In addition, companions provided emotional support to delivering women through encouragement and reassurance. Only one participant expressed indifference towards the presence of her companion, while the majority believed companions provided the courage and strength necessary to give birth. The mere presence of a companion was comforting as it promoted a feeling of belonging. Such support might even aid vaginal delivery, as several participants speculated that they might have had a caesarean section had a companion not been around.

*Even not doing anything but seeing her around*,* that one alone is a great thing… that shows that I belong.* Delivering Woman, IDI6, age 29.

*If they were not allowing*,* (…) maybe I would just be discouraged*,* give up. Maybe they should go and do operation*,* because I was really afraid.* Delivering Woman, IDI9, age 23.

Delivering women related differently to companions than to HCWs and expressed gratitude that there was someone attentive to their needs exclusively. Companions had a unique capacity to reassure patients because, unlike HCWs, they were familiar to the patients. Certain requests that would appear inappropriate when presented to HCWs could be suitably relayed to the companion.

*[The companion] can obey you more than the nurses (…) because you can tell her*,* come*,* rub my feet! Cover me! Take it off! They can do that. But you can’t say that to a staff.* Female Companion, FGD7.

As companions were familiar with the delivering women, they could provide insights to staff on how to encourage and reassure them. Companions could also suggest innovative ideas, such as one companion who developed a tug-of-war mechanism to promote pushing. Patients, particularly primigravida women, tended to cooperate more in the presence of their companions.

### Disadvantages of companionship

However, some patients became less cooperative with staff when accompanied. HCWs reported that some patients listened more to an older relative or a TBA than the HCWs, leading to noncompliance. Companions themselves might mistreat HCWs or the delivering women, even encouraging or engaging in physical and emotional abuse.

*The patient will accept their own view more than the midwives or nurses. Because they will know the birth companion*,* the relatives*,* maybe their sisters. Especially the females*,* or the big sisters. Especially those TBA… Yes*,* they will listen to their word more than (…) the nurses.* Healthcare Worker, FGD2.

*Some of the birth companions even ask us to flog the patients (…) ‘Nurse*,* don’t joke with her. Flog her! She will lie down and deliver. If you don’t flog her*,* she will not be cooperative.’* Healthcare Worker, FGD5.

HCWs noted that privacy concerns remained an issue. Some companions might be overly curious about neighboring patients, or they might discuss details of the birth with others in the corridor, violating the patient’s privacy. This was especially an issue when a patient required emergency treatment or assisted vaginal delivery.

At times, HCWs had to protect the privacy of the patient from her own companion. Due to stigma, HIV-positive patients might not divulge their status to relatives, so it was up to the HCW to hide the patient’s status from the companion. Without a private moment between HCW and patient, the HIV status may not be disclosed, putting the HCW in a position of risk.

*The fluid spilled on my skin when I was doing delivery (…) so I went straight to take this kit*,* this kit to do this test. So I did it and she was positive… So I asked her*,* I said*,* did you know that you are positive? She say*,* yes (…) Then I ask her*,* why didn’t you tell me before? She said sorry*,* she was apologizing to me*,* ‘I am taking my drugs*,* please*,* I am sorry. But please don’t tell my birth companion.’* Healthcare Worker, FGD5.

Birth companions sometimes caused extra work for HCWs. Occasionally, companions might faint from the sight of the birth, so the HCW would have to attend to both companion and patient.

Concern was also raised about interference. Some companions adjusted the IV line to speed up the administration of a medication. Others encouraged the patient to eat or drink despite medical instruction, such as immediately prior to an operation, or provided native herbs to the patient without consulting the HCWs, which could cause adverse interactions with medications. Companions might also dangerously encourage the patient to push before she is ready, lie in an incorrect position, or use the toilet when the baby is already beginning to emerge. HCWs reported that due to this interference, they felt responsibility to monitor the companions.

*When they are in there (…) you should monitor them (…) because they will do what they feel like doing*,* which is not good for us and the patients. That’s why when they are there*,* we don’t leave them all by themselves.* Healthcare Worker, FGD2.

Educating the companions and ensuring that they are aware of boundaries was deemed necessary to prevent inappropriate interference with their work.

### Companionship and the community

BCC may also strengthen the trust between community and healthcare system. Many companions expressed that they better understood FBD through witnessing the birth.

*I was there watching*,* what is this name? What is they go for use? What does this thing mean? So I ask the doctor*,* they say*,* OK*,* so this what this thing mean… I was asking about what they are doing… I want to get more idea.* Female Companion, FGD11.

Several participants reported that they shared their positive experiences with others in their community, encouraging them to give birth at PCMH.

*Even when I’m at home*,* even my neighbor*,* I even told her*,* I said*,* please my dear*,* when you are in labor*,* come to [PCMH]. Here the nurses and the doctors are very nice. They encourage the patients (…) I said*,* please don’t sit at home and give birth*,* come to the hospital.* Delivering Woman, FGD3.

HCWs expressed relief that companions were providing correct information to the community and reported feeling more appreciated since implementing BCC.

*It’s also boosting our morales*,* that we are doing a good job… They go outside and say PCMH is very good*,* go outside and bring your patient*,* so we feel proud.* Healthcare Worker, FGD5.

Both companions and delivering women expressed satisfaction with the treatment from HCWs. However, some participants reported negative interactions with the medical staff, citing rudeness or arrogance.

When asked if they would prefer FBD or home birth for future deliveries, participants overwhelmingly preferred FBD. In one exception, a woman expressed that, barring any complications, a home birth would be preferable due to increased freedom. Overall, FBD was associated with fewer risks and improved care.

*To deliver in the hospital is very fine. Because when you deliver at home*,* they have complication at home. But when you came to the hospital*,* the doctors are here*,* the nurses are here*,* so all of them they will come together to see that you deliver safe.* Delivering Woman, FGD3.

## Discussion

This qualitative study explored the perceptions of individuals experiencing BCC in a setting characterized by extreme poverty and high maternal mortality. The results support the existing evidence for BCC as a key strategy to promote a positive childbirth experience and strengthen the relationship between community and healthcare system.

### A positive birth experience

Nearly all the women in our study, as in previous studies [17, 19], expressed desire to have a companion of choice during birth and satisfaction with the presence of a trusted companion. That companions support delivering women by taking care of practical needs, dispensing guidance and clarification, advocating for the patient’s needs, and providing emotional support and comfort is already well-established [15, 19]. On an interpersonal level, it was emphasized that a companion could provide support in a way that HCWs could not, due to both the familiarity of the companion and the formality of the person-provider relationship. Similar findings have been noted in other facilities [22], suggesting that the presence of a companion may “fill in the gaps” in interactions between the delivering woman and HCW.

While other studies [15, 17, 19] found that women overwhelmingly preferred female companions, several participants in our study posited that male companions could provide more emotional support than their female counterparts. Complying with results from Uganda [34], participants felt that male participation during birth increased familial bonding and made women feel more appreciated. A novel finding of our study is that both men and women believed that male participation during birth could improve the treatment of women afterwards, as the man would develop a heightened appreciation for a woman when witnessing her pain and struggle. This points to the general underlying societal gender inequity and the undervalued position women face in their maternal role. Although including male companions into the birth process might be logistically more challenging (e.g., ensuring privacy for other delivering women), our findings indicate that it might be especially important to enable male birth companionship in a cultural context where women usually receive less social recognition.

Leinweber et al. [35] define a positive childbirth experience as a woman-centered experience that allows her to feel supported, in control, safe, and respected. At PCMH, women felt supported by the presence of their companion; practical assistance and increased access to information promoted a feeling of being in control; the presence of someone familiar as a witness allowed women to feel safe; and women felt respected as they had somebody to take care of their needs. Other studies [5, 14] have shown that women who have continuous support during birth are more likely to experience respectful maternity care and less likely to receive mistreatment from medical staff [11, 12, 36]. The participants in our study intuited these findings and felt protected by the presence of their companions. However, it is important to address that in some cases, companions were encouraged by HCWs to enact abuse, by physically forcing the woman’s legs open during birth. This unacceptable act of physical restraint is a type of mistreatment during childbirth [37] and begs further research on how to ensure that BCC limits abuse rather than enhances it.

An improved childbirth experience through BCC has the potential to increase FBD. Many studies [4, 38, 39] have shown that perceived quality of care has significant influence on acceptability and utilization of FBD. While the women in our study, as in previous studies [23, 40], were aware of the reduced risk of complications associated with FBD, they also expressed apprehension of mistreatment and fear of being alone. These findings indicate that FBD awareness campaigns alone are insufficient to increase the number women delivering in facilities and that efforts to improve perceived quality of care are critical. While BCC is only one component of fostering a positive birth experience, our results suggest that it is not only a simple and cost-effective way to dramatically improve women’s perceived quality of care but should also be considered a universal human right as much as respectful maternity care [7].

### Practicing companionship

The implementation of BCC at PCMH, much as in other settings [41], is influenced by the dynamic values and decisions of the HCWs. BCC was only regularly practiced at PCMH after enforcing training, and HCWs reported that this institutionalized encouragement is what allowed the change in behavior and attitude towards companionship to take place. As in other studies [21], societal norms and culture was a barrier to implementation of companionship; as their seniors discouraged companionship, younger HCWs had no reason to question them, despite having been exposed to the idea of BCC in midwife education. This suggests that rigid hierarchy in the professional medical environment may impede good practice. Other institutional barriers to companionship, such as concerns about privacy and interference, support findings from previous studies [15, 17, 19, 26, 42].

This initial reluctance was juxtaposed with an enthusiasm for BCC expressed during FGDs and IDIs. HCWs detailed the many advantages of companionship for their own work, including a reduced workload, confirming findings from Kenya and Tanzania [19, 25]. While other studies have found that HCWs’ fear of litigation was a barrier to companionship [15, 26], the HCWs in our study felt protected by the presence of a third party as witness and unanimously expressed that companionship alleviated their fears of unwarranted allegations.

Despite several challenges linked with BCC, it is encouraging to note that HCWs described finding solutions to these challenges, such as educating a companion about the boundaries of their role upon entering the labor ward. Other solutions, such as rearranging the ward so that there is sufficient space and privacy, may require some further logistic and financial support, and it would be desirable that decision-making bodies for health service planning and quality of care within the MoHS prioritize this aspect in the future.

### Fear and mistrust

Multidimensional fear and mistrust were relevant themes on different socio-ecological levels. Interpersonally, distrust between HCWs and delivering women has been described in other settings [43]. Lack of trust in a companion was also a potential barrier to companionship, such as in the case of HIV-positive patients. Concern about witchcraft has been reported in Malawi [15] as well as among participants in our study.

On a community level, HCWs reported being frequently accused of stealing or exchanging babies, and other participants confirmed the presence of these rumors in the community. This resembles rumors spread about healthcare services during the Ebola outbreak of 2014–2016 [44] and suggest that fear is a significant and historically-grown barrier to FBD in Sierra Leone. This mistrust of the healthcare system is not an isolated phenomenon, but a grave consequence of colonialism and centuries of violence [45]. While a detailed analysis of these forces is beyond the scope of this study, mistrust can be understood as a valid critique of colonial legacies, rather than simply the absence of trust [45]. BCC, by centering the woman’s experience, may strengthen her position as a rights-entitled individual and alleviate these feelings of mistrust.

Our results suggest that companionship might increase trust between PCMH and the community. Participants expressed greater satisfaction with their perceived quality of care when companions were present and reported encouraging others in their community to deliver in hospital. Nuriddin et al. [46] found that during the Ebola outbreak in Sierra Leone, trust in HCWs was influenced by perceptions of the quality of patient-provider interactions. At PCMH, participants enthusiastically expressed that they would deliver at PCMH again in the future because of their positive experiences. Companions also reported learning more about FBDs, resembling findings in Malawi [15]. While Banda et al. [15] expressed concern that companions might feel emboldened to assist in deliveries beyond their capabilities, participants in our study were impressed with hospital care, increasing exposure to facility-based care and potentially alleviating fears.

### Strengths and limitations

This is one of the first in-depth assessments of BCC in Sierra Leone. A limitation is the restriction of our study to only one urban health facility. Further assessments should examine how BCC is received in rural settings and different facility types, as well as include the unique perspectives of marginalized groups, such as underage women. Also, invitation by telephone to participate in a study was sometimes perceived as unusual. Therefore, it is possible that only individuals who were trustful of the healthcare system were willing to participate, potentially causing selection bias. However, our results suggest that participants were not hesitant to express critical opinions. Another possible limitation is that, due to the unpredictability of the rainy season and the challenge of securing reliable transportation, participants arrived at PCMH at irregular times. In effort to avoid unreasonable waiting time for participants, the size and composition of the FGDs had to be determined spontaneously, which resulted in four dyadic FGDs with only two participants. This might have led to less exchange of opinions among the participants. However, we adapted the interview style according to group sizes to make participants feel welcome to share their insights regardless of the group composition. As a qualitative study, one limitation is the influence of the researchers’ characteristics on the research process, possibly impacting the study’s transferability and objectivity. We believe that this personal involvement is as much a strength as a limitation, as, when combined with rigorous reflexivity, it can lead to richer insights and a more empathetic understanding of participants’ experiences.

### Implications for practice

Our findings support the implementation of BCC as a strategy to increase women’s autonomy and choice during childbirth, provide protections for both HCWs and women, and strengthen the relationship between healthcare systems and the community by increasing exposure and appreciation of facility-based care. Prioritization of addressing barriers to BCC, such as arranging the ward to have sufficient space and privacy, are important policy considerations. Supporting women’s autonomy and choice includes encouraging and supporting male as well as female participation in the childbirth process. While BCC generally supports the rights of women, care must be taken to ensure that companions are not recruited by HCWs to engage in mistreatment of women.

## Conclusion

In our study, women delivering with BCC detailed numerous ways in which they felt supported by their companion. Their experiences suggest BCC is a key component of a positive birth experience and may promote FBD. BCC presented new challenges for HCWs, but HCWs described several strategies to deal with these challenges and emphasized the benefits, such as improved workload and job satisfaction. Both HCWs and women felt protected by the presence of a third party as witness. Companions themselves reported an increased understanding and appreciation of facility-based care.

In conditions of scarcity and mistrust, our study suggests that BCC is not only a valuable tool that should be scaled-up to strengthen perceived quality of care and relations between community and healthcare system, but a means of empowering women to realize their rights to autonomy, privacy, and security.

## Electronic supplementary material

Below is the link to the electronic supplementary material.

**Supplementary Material 1**: **Additional file 1**. Evidence for identified categories, themes, and sub-themes. A supplementary file containing direct quotations to support the themes and sub-themes presented in Table 1.

## Data Availability

Anonymized transcriptions of the FGDs and IDIs are available from the corresponding author on reasonable request. Direct quotations supporting the themes and sub-themes in Table 1 are available as an additional file.

## Abbreviations

MMR: Maternal mortality rate
FBD: Facility-based delivery
BCC: Birth companionship of choice
HCW: Healthcare worker
PCMH: Princess Christian Maternity Hospital, Freetown, Sierra Leone
MoHS: Ministry of Health and Sanitation, Sierra Leone
FGD: Focus group discussion
IDI: In-depth interview
