# Supplementary material for: “It shows that I belong”: a qualitative study assessing birth companionship of choice in a maternity clinic in Freetown, Sierra Leone
Source: BMC Pregnancy Childbirth. 2025 May 27;25:618. doi: 10.1186/s12884-025-07711-z (PMC12107989; doi:10.1186/s12884-025-07711-z)
Supplement: Supplementary file 1 — Supplementary Material 1: Additional file 1. Evidence for identified categories, themes, and sub-themes. A supplementary file containing direct quotations to support the themes and sub-themes presented in Table 1. [file 12884_2025_7711_MOESM1_ESM.pdf]

“It shows that I belong”: a qualitative study assessing birth companionship of choice in a maternity clinic in Freetown, Sierra Leone

Additional file 1 – evidence for identified categories, themes, and sub-themes

Types of support provided by companions 3

Instrumental support 3
Gathering supplies 3
Hygiene 3
Food and water 4
Assistance with baby 4

Informational support 5
Medical reminders 5
Advice and guidance 5
Communicate with relatives outside 6

Advocacy 6
Companion can call for help 6
Protection from abuse 7

Comfort 8
Physical touch 8
Prayer 9
Physical comfort 9
Familiarity 9

Emotional support 10
Encouragement and strength 10
Companion as proxy 13

Choosing a companion 13

Male vs. female companions 13
Better treatment from husbands after witnessing birth 13
Empathy and emotional support 14
Increased bonding, unites the family 15
Men are afraid 15
Women provide more expertise 16

Trust 17
Fear of witchcraft 17
Trust by giving good advice 18
Trust to be non-judgmental and confidential 18
Trust by sharing everything 19
Trust by support during pregnancy 19

Implementing companionship 20

Reasons for previously not allowing companionship 20
Concerns about privacy 20
Threat of theft or scamming 21
Lack of space and resources 21
Arguments with relatives 21

Educating the companion 22

Monitoring the companion 23

Knowledge of importance of companionship 23

Seniors were not implementing 23
Lack of knowledge 23

Workshop, institutionalization means easier implementation *24*

Satisfaction with companionship *24*

Advantages for nurses **24**

Reduced workload *24*
Companions help physically during labor 25
Companions can fetch medical supplies 25
Relief from providing emotional support 26

Communication *26*
Language barriers and translation 26
Companions provide reliable information 27

Companion as witness *28*
Protect nurse from false claims of abuse 28
Protect nurse from false claims of baby theft 28

Easier to deal with patients *30*
Companions know the patient best 30
Patients become more cooperative 30

Disadvantages for nurses **33**

Inappropriate behavior *33*
Encourage or engage in physical and emotional abuse 33
Companion makes the patient less cooperative 33

Interference *34*
Interference with medical processes 34
Native herbs 35
Dangerous encouragements 35

Privacy *35*
HIV and stigma 35
Companions curious about other patients 36
Companions talk outside about what they witnessed 37

Extra work *37*
Extra materials necessary 37
Companions faint and require attention 37

Companionship and the community **38**

Home vs. hospital delivery *38*

Companion is learning about FBDs *39*

Treatment from nurses *39*
Satisfaction with treatment 39
Mistreatment and abuse 40

Sharing about experience with community *40*
Companions give accurate information to community 40
Encourage FBD in the community 41

Types of support provided by companions

Instrumental support

Gathering supplies

She would come with food for me. Yes, she would even help me with the baby... even to wear clothes, yes, she would come with cloths, she would come with pants, she would help me with all these things that I need. Food, water. She was a great help to me. *Delivering Woman, IDI6, age 29*

So my husband went home to collect the cloth for the baby, because we did not bring anything like that. *Delivering Woman, FGD10*

I was not told that I should come with soap at the labor ward. So when I come here, I didn't have soap and other things, so she went out and buy them. *Delivering Woman, FGD6*

When the child is born, they will ask you to come with clothes – *lappa*. So if you are unconscious, you won't be able to provide that cloth. But with a companion by, she will be able to get the nurses things. *Delivering Woman, IDI6, age 29*

She leave me inside, and she went out to run buy some things for the baby, like, diaper, wipes, baby clothes. *Delivering Woman, IDI3, age 23*

Some birth companion, if we ask for anything, they will go outside and get it for the patient. Maybe we ask for a pad, the patient doesn't have any pad, the birth companion will go and get the pad for the patient. The *lappa,* the different items for the patient. *Healthcare Worker, FGD5*

The nurse lady speak to me, say you have to come with soap, *lappa*, pampers. So then at that time I went outside, buy those things. Then I come. *Companion, IDI4, age 27*

So when I went to the labor ward, they asked me to bring items like the *lappa*, the baby cloth, the plastic for the bed. And I brought all those things. *Companion, FGD7*

At that morning, I went for some pads, because this girl used many pads. I go and bought and collect some pads. I bring them there. So she go and take bath, both of us go and take bath in the bathroom. *Companion, IDI10, age 26*

Hygiene

She was there packing all my things. Yes, packing all of my things. Yes the one clothes with blood, so she was packing it inside the plastic, she was there to take care of it. *Delivering Woman, IDI11, age 32*

Clean her up properly, take the dirty linen home. We give it to her mother so she can go and clean the dirty linen… Because water, water is very difficult, there is a challenge of water. *Companion, FGD4*

I help them clean, even the other nurses came and help me clean... I take her to the bathroom, to help her clean. *Companion, FGD11*

I would want my mother-in-law to be there, because my mother-in-law will be of help to help her for like laundering the baby's stuff or my own linens, and my mother-in-law can also be helping me to carry the baby around. *Delivering Woman, IDI5, age 19*

I be get somebody near me when I deliver. They clean the thing, my sister will clean everything. Then you take water, you go to the bathroom... I go wash, I come and dress. *Delivering Woman, FGD3*

Food and water

When the relative is around, if you want food, the relative will go out and look for the food for you and bring it. It's simple. *Healthcare Worker, FGD2*

Giving her water if she needs water, even food. Especially the first stage of labor. See, the relative will even assist you to feed the pregnant woman. If she request for food, they will give her, because she needs energy to push that baby. *Healthcare Worker, IDI8, age 50*

So even the very day on her delivery, I went out to buy this *maltina* for her to drink. So I went out and bought the *maltina* and I gave her to drink. And some other stuff like food, because the first thing that she asked for was *foo-foo*. Do you know *foo-foo*? Yes, so that was the first food she asked for after delivery. Then I went out, go in search of it, then I bought it and bring it for her to eat. *Companion, IDI4, age 27*

'Go and buy *maltina,*' they said, 'go out and buy *maltina* for her.' She went out and buy *maltina* for me. I was so weak. My body was all over nervous. 'So go out and buy *maltina* for her.' But after that, when I drank the *maltina,* I was so strong. *Delivering Woman, FGD6*

At that time, I call on my sister-in-law to get a flask so that she can have a bath. Then she came with it, we used the hot water. But at that moment, she said she want to eat, I'm hungry. So I went downstairs, buy food for her. She ate. *Companion, FGD11*

When they want anything, they send their birth companion. They tell them, when some want to drink water, 'can you go buy water for me?' And the companion do that for them. *Healthcare Worker, IDI2, age 40*

At that moment, since it was CS and you know, it is not easy for you to get over that time, so she will help you doing some work like she will fetch for you water, bring food, take care of you, all of this stuff. *Delivering Woman, FGD6*

They help in giving the water, if they want to drink water… They feed them at times. *Healthcare Worker, FGD1*

I talk with her, she said she want to eat something. I went to the street and buy it, got some food for her, some drinks or water. Yes. *Companion, FGD4*

They go get food for me. I eat. My mother made food for me, she got it. *Delivering Woman, FGD3*

Assistance with baby

After the delivery they will keep my baby outside, my sister was also with them. So go there and weigh, to know the weight of the baby. And they ask my sister to go and buy that wiper, because I did not come with wiper. Wiper and baby oil to rub it on my baby. *Delivering Woman, FGD10*

She will help to dress up the baby. Yes, at times. They will help us, because we are trying to see about the mother. Then the birth companion, they will wipe the baby for us, dress them, wrap them for us. They will put them on that warmer. *Healthcare Worker, IDI2, age 40*

I started to wash the baby, oh the baby is very nice. So on that day I feel good. *Companion, IDI13, age unknown*

She really helped me. Because at that point she was the one who was going the extra mile to get me food, to get me water to drink, and even at the period when I have given birth to the baby, she was the one like laundering the baby's clothes, laundering my own clothes. She was the one who was pampering the baby, because I was not – because it's a CS case, I will not be able to carry the baby just like that. So my mother-in-law was the one carrying the baby for me. *Delivering Woman, IDI5, age 19*

After I delivered the baby, my sister was the one carrying the baby. She helped me to like, dress the baby, carry the baby, clean the linens, my personal linens. Those are some of the other things my sister did. *Delivering Woman, IDI12, age unknown*

At that time you won't be able to carry the baby because it's CS, so she will be there carrying the baby. And help you to breastfeed the baby. When you finish breastfeeding the baby she will carry the baby again. *Delivering Woman, FGD6*

Informational support

Medical reminders

The doctor asked me to be one with her. Let me now allow her to sleep... because the very day, I don't know. They said after delivery, they don't allow her to sleep. So let me talking to her while she is recovering from high fever. *Companion, IDI4, age 27*

I was there because she called, I stood by her. She was crying for water and I don't want her to take water because she was like, to be on fast until after the operation. *Companion, FGD7*

If the wife forgets to do the sit-bath, the husband will remember... So it's really nice, because if you're alone, you will just skip something and say, I will do it later. But if you have someone asking you, 'have you done this?' Then you will say, 'OK, I should do it now.' *Healthcare Worker, FGD1*

There should be a next of kin... to take care of the patient, so in case maybe the patient doesn't forget what the medical practitioner tells her... She will be there to remind her in order for everything to be good about the patient. *Healthcare Worker, FGD1*

Advice and guidance

I give her comforting words, because she was complaining her stomach. So I just explained to her that it's normal... I decided to explain to her that everything is contracting. *Companion, IDI4, age 27*

I ask my mom, 'mom where is the baby going to come out? They are going to open my stomach to remove the baby?' My mom said, 'where that thing entered is where the baby is going to come out.' I said, 'eh?' They said, 'yes, when the time for delivery, you will know that time. That time

you will know where the baby is going to come out.' So you see? They encourage me. *Delivering Woman, FGD3*

When I am crying, the pain, the labor pain, my mother-in-law used to tell me, 'please take her, you have to bear the pain. You know, you are a woman, I am a woman, this process or stage you are going through is where all of us came from. So you will definitely be fine.' *Delivering Woman, IDI5, age 19*

Communicate with relatives outside

My sister, she was giving me good information about my wife. So I was taking courage. *Companion, FGD4*

A companion should be there as like an intermediary between the healthcare workers and the relatives. They appreciate it. *Companion, FGD7*

The mother-in-law was communicating with my husband. It's helpful, because my mother-in-law related the information to my husband, it kept my husband in an ease position, that to say my wife is OK and the baby is fine, they are doing OK. *Delivering Woman, IDI5, age 19*

And the companion can even call the relatives. They can communicate... I was there to communicate, to the husband, to the grandmother, to tell them, 'ah we are about to go to the operating theatre but now they have an emergency so we have to wait a bit.' *Companion, FGD7*

Advocacy

Companion can call for help

After some time, when labor progress, the pressure shoot up. So I have to call the other doctor. *Companion, FGD4*

They communicate with them, the nurses who came into the labor room. Because at that time they give me some drips, my sister will ask them, 'what is this drip for? What is this injection for?' So they interact. *Delivering Woman, IDI9, age 23*

Because when they're in the room with the patient, if the patient wants to remove IV, they will call us, 'nurse, please come.' They will call us, when the patient refuse to lie on the bed. *Healthcare Worker, FGD5*

Your companion will be there talking to the nurses, so that particular person will be able to take care of you, will be able to give you pill or medication, or have concern about that... if you don't have companion with you, during the birth or right after giving birth, things will not go normal, because the nurses have plenty people to be taking care of. But if your companion is there, she will be there, talking to them, talking to them, go there, see about this, and talk to her. *Delivering Woman, FGD6*

Then I told them, when the IV fluid was almost finished, I have to inform. I say, 'look, I think this thing is about to finish.' They said yes, so they changed it to another one that was prescribed. *Companion, FGD7*

They did not communicate, unless my aunt is the one that go and find the doctor... They go and find a doctor, tell the doctor that you have a patient that is waiting for you. *Delivering Woman, FGD6*

My husband was there all through the time. Only when I started feeling my waist heavy, that's the time my husband told the nurses, then they took us to the labor ward. *Delivering Woman, FGD8*

When I was crying, she will say that, 'nurse, go and look at my sister! She is crying!' The nurse will go there and look at me. *Delivering Woman, FGD6*

I will sit and write my notes. The birth companion will stand at the patient, he will be know, if something will want to go wrong, she will call me, 'nurse! Something is going on!' So I come and see. *Healthcare Worker, IDI2, age 40*

My aunt signed the consent form. Because I was admitted on Thursday, and I went through the caesarean on Friday. So she signed it on Thursday. *Delivering Woman, FGD6*

If my mother-in-law is present, the nurses will communicate with my mother-in-law. But in the absence of my mother-in-law, they will communicate to me directly. *Delivering Woman, IDI5, age 19*

When there is a birth companion, if you have delivered, maybe you have finished all what you have done for the patient. She'll be stand now and wash that patient. If she get dizziness, she will call you to attention. This patient is getting dizzy now, you will come and give her treatment. *Healthcare Worker, IDI2, age 40*

Some [birth companions] will even go to talk to the nurses, whenever they see any sign, like any strange something. Like for example, maybe the nurse has gone to chat. The relative is with the patient. Whatever sign or things that relative sees, they will be calling us to attention, quickly. *Healthcare Worker, IDI8, age 50*

Maybe the nurse is with two patients, she is supervising two patients, and you are not with that patient because you are doing the partogramm, maybe you are monitoring another case. Then the relative is inside. And that patient is having, let me say, fitting. He or she will call you quickly. Whatever you are doing, stop doing and assist. *Healthcare Worker, IDI8, age 50*

Protection from abuse

It is important for the mother-in-law to be in there. That will make me feel at ease. But if the mother-in-law is unable to be there, I would not feel happy, because I will be afraid... I wouldn't have somebody that would observe what is happening in there... I will be happy if my mother-in-law can be observing how they will be treating the baby, maybe they will not give any bad treatment to the baby, like exchange the baby or so, and also my mother-in-law could be watching how they are treating me, so when I am back from the experience, they can tell me exactly what happened. *Delivering Woman, IDI5, age 19*

It will be good that you stay with your family during the time of delivery. Because you will deliver, they took the baby, you will see, nothing to hide. You will see everything. *Companion, IDI13, age unknown*

And the patient is also happy the birth companion is in there, seeing everything that is going on. *Healthcare Worker, IDI1, age 36*

To be honest with you, these nurses, some are nice, some are arrogant. So to use the words. Because for some, you ask them for help, to be honest with you, they will. But some, when you ask them, before giving you and helping you they have to complain, say two three words that will not be favorable onto you. So that's to be honest with you. So my sister by my side, I don't have any problem with nurse. *Delivering Woman, IDI6, age 29*

Everything was OK by now, because they are around me. Especially when they offer prayer. *Delivering Woman, IDI9, age 23*

I'm standing there, just to protect my sister when she is in labor. *Companion, IDI13, age unknown*

She ensure that I was taken care of... After the delivery, they use the soap to clean me up, yes, and they mop the floor. So she was there to see that all these things were done, though she was not the one that did it. *Delivering Woman, IDI6, age 29*

If you have a companion they treat you differently, they treat you good. Because they know that the companion will – the companion will talk to them, like my aunt... So they treat you well, because you have someone in there. *Delivering Woman, FGD6*

With my presence there, nobody would insinuate her or try to use the women's rights the wrong way. Like, you have to push to bed, we the nurse didn't tell you to get pregnant. But with my presence there, I was providing the right energy and the right words for her to give birth. *Companion, FGD9*

If you have a companion there, if the nurses are bringing negative energy, your companion will serve as your shield and your protector to the nurse, because some nurses are aggressive, you know? *Companion, FGD9*

Even the nurses, if they have some negative energy that they want to bring up, they will say, 'no, she is with somebody.' Even if she cannot fight us back, but her companion can fight us back. *Companion, FGD9*

Comfort

Physical touch

Another thing that the relatives do is, they give a sacral massage. *Healthcare Worker, FGD1*

She was there, holding me, touching me, just be patient. *Delivering Woman, IDI11, age 32*

I was feeling pain and she was encouraging me, massaging my feet, rubbing my hand. *Delivering Woman, FGD6*

I was sitting close by her, massaging her, rubbing her head, giving her the courage that everything will soon go on. *Companion, FGD7*

Some will stand by the side of the patient, and talk to her and encourage her, rub her back. *Healthcare Worker, IDI2, age 40*

I will hug her, touch her, I kiss her too. I say, take it easy, please. *Companion, IDI10, age 26*

I started shivering like this, then my husband started to warm me up like this. Praying for me, told me some inspiration words, I love you baby. *Delivering Woman, IDI3, age 23*

She was delivering inside the delivery room, she was holding my hand, and the doctor was trying to tell her to explain to her that let her push. *Companion, FGD11*

Prayer

She was just praying... so that it will be simple and be soft for me... I feel good, because I really felt good by then... That time when your family member, friend, or loved one is in there, you have to pray for that person. Because it's only through the grace of god. That is it. She was praying. I really felt good having her by my side. *Delivering Woman, IDI6, age 29*

Prayer for her, then I console her... I said, 'believe god. Don't lose faith. With god all things are possible.' *Companion, FGD4*

I started praying for her. Immediately I started praying, she saw progress in the delivery stage. *Companion, FGD9*

Physical comfort

So she go and take bath, both of us go and take bath in the bathroom. *Companion, IDI10, age 26*

During the period for medication, you know the cuts, it's difficult for me to get up by herself. So my companion was the one who can like, raise me, to say like, please be seated, your medications are here. *Delivering Woman, IDI5, age 19*

To help her, carry the child, like if the parent want to go and take a bath, help her with water. *Companion, FGD7*

Familiarity

It is so good to have a companion, when you have somebody to talk to. Talking to you, encouraging you, you have the courage. But if you don't have anybody – because the nurses will not encourage you. 'Hey! Make like this! Go this way!' They will not encourage you like the way you have somebody that will encourage you, say prayer for you. They will just shout, 'do you want to kill the baby! Open your leg! Do like this!' They will just be like that. So you know, you will be scared. *Delivering Woman, FGD6*

The individual will help you and stand by you. They can obey you more than the nurses... because you can tell her, come, rub my feet! Cover me! Take it off! They can do that. But you can't say that to a staff. *Companion, FGD7*

You know, the nurse, they can't buy it for me. But if you there with somebody with you, you will say, 'now go buy it for me, I want it. So go buy water for I want to drink water.' *Delivering Woman, FGD3*

The nurse is there to do her own duties but the birth companion is there to support. *Healthcare Worker, FGD1*

If I was alone, maybe I will not feel fine because I don't have anybody to console me, because the only person that's in the labor room is the person who's trying to help me to deliver the baby... I don't have that person to say, 'hey, come and rub my leg.' Even the doctor, I will never tell her to help her to rub my leg. She will say, 'eh, am I the one that give you the baby?' *Companion, FGD11*

That individual will be there just for my own service. 'Give me water! Rub my head! Rub my eyes!' They can do that. *Companion, FGD7*

If I have something, I want to go and take money, I will say, 'look, my aunty, go and take money, I want to do something.' Or maybe I don't have somebody to send. I cannot tell the nurse, they have work in the hospital. *Companion, IDI13, age unknown*

It's better to have somebody with you because when you have somebody you know, it will give you courage. *Companion, IDI13, age unknown*

When it's your relative by you, you feel safe at that time. Your relative is by you, hugging you, massaging you. You don't know the nurse, you are just knowing the nurse for the first time. *Healthcare Worker, FGD1*

In the time of delivery you need to console the patient. So by consoling her, you need to have someone close to her or close to her heart. By doing so, then the doctor will do so successfully. *Companion, IDI4, age 27*

The nurse is a complete stranger. Because some of them we are meeting them for the first time. But if somebody that you have known who is the birth companion, it's always better to be there. *Healthcare Worker, FGD1*

Emotional support

Encouragement and strength

I was very comfortable with my husband in the labor room... Because I don't have anyone to console me, he was the only person that was able to console me when I was giving birth. *Companion, FGD11*

She was there encouraging me because, you know, at first birth was difficult. Yeah she was saying it would be easy, encouraging me. Even if I want to give up, she said you have to try, you have to push on. So they encouraging me... She was there encouraging me, asking me to try, 'that is it, you have to go through it, you have to try, do your best for you to be safe and your baby to be safe.' *Delivering Woman, IDI6, age 29*

When you are delivering, if you have someone with you, it gives you courage to do the work. So when I gave birth here in this hospital, when my sister is there, I was so having encouraged. They are praying for me, giving me courage. *Delivering Woman, FGD6*

My companion was providing me with a lot of courageous words like giving them hope to say, 'nothing is going to go wrong with you. You're going to put to bed safe.' *Delivering Woman, FGD3*

I choose her because I want to feel free, and be comfortable of myself. Because I don't want to be too frightened. When I see them around me, it ease my stress... when I was in the labor room

they encourage me, when I was crying, they courage me, tell me to calm down. So I was a little bit easing my stress. *Delivering Woman, IDI9, age 23*

During those times, you have to have somebody by you. They encourage you. Yes, even seeing them around, you will feel motivated. Yes, seeing your companions, your people around. You feel motivated. But, no one, you feel neglected. Say, am I not important? At this time, I need these people and they are no even showing up. You feel neglected and feel as if you're outcast. So you see, that is it. *Delivering Woman, IDI6, age 29*

Even not doing anything but seeing her around, that one alone is a great thing. She is very appreciative of me... that shows that I belong. That I belong to some people, yes, that people will need me, that is it. That I belong. When I was there, other people came around, I really felt good, I felt important. Now I know that I'm important, people love me. *Delivering Woman, IDI6, age 29*

While my sister was here, the presence of the my would give me confidence and zeal to push through the procedure. If my sister were not here, I would have been feeling discouraged. I would not have the might or the zeal to do it alone. *Delivering Woman, IDI12, age unknown*

We had one case, it was a multigravida case but the labor was not progressing... So the husband calmed the woman down, because we tried to talk to her but she was crying , because of the fears... But the husband calmed her down. *Healthcare Worker, FGD2*

You will just sit by them, talk to them. You can courage them, if you want to eat you can eat so you will have strength to push. Some will refuse, say, 'I don't want to eat.' But you have to talk to them, say, eat. *Companion, FGD4*

It's good for somebody to be by your side when you're in labor, because the presence of that individual by your side, it gives you courage, it gives you strength, and supports you. *Companion, FGD7*

You be stressed, the mother, it will turn up the pressure. But if you have someone who encourages you, I think you don't have pressure. You have someone there that encouraging you. *Delivering Woman, FGD6*

When she was like crying, the pain, I was comforting her. 'Please bear with it...' I was talking to her as well, 'please be calm, it's going to be fine.' Like, providing words of courage or comfort. *Companion, FGD9*

They said if they don't see any signs and symptoms, they will take me to the theatre. I started crying, I started praying. Crying, praying. My sister said, 'the devil is a liar! You are going to deliver yourself.' *Delivering Woman, FGD6*

I'm afraid of labor. Because of that kind of pain, the way I feel, I don't think if there was no one there with me, I would have been able to do it. *Delivering Woman, FGD8*

If my husband was not there, I would not have been able to deliver. So you know definitely it would have led to a CS. *Delivering Woman, FGD8*

If they were not allowing, I would just, maybe I would just be discouraged, give up. Maybe they should go and do operation, because I was really afraid. *Delivering Woman, IDI9, age 23*

When the pain is getting worse, I want to say, 'please take me to the theatre, let me go and do operation.' And my husband and sister say, 'no no no, don't say that, you can do it. God is with you.' Yeah. Because I want to say, 'please take me to the theatre, let me do operation, I have suffered too much.' And my sister and my husband say, 'no, you can do it.' So I feel good. It's better when they were around me. *Delivering Woman, IDI9, age 23*

Later my sister was so scared. I said, let me talk to her... so I talk to her, I give her encouraging words. *Companion, FGD4*

So I have to give her the courage that she has to wait, very soon she will hear the cry of her baby, you know, I give her all these positive vibes. *Companion, FGD7*

When I was in pain, she just encouraging me, said take it easy, that's all about giving birth, she was there encouraging me, said just be patient, when the time comes, you will deliver your baby safely. *Delivering Woman, IDI11, age 32*

Even though I was feeling pain, I knew that there was somebody giving me encouraging words. *Delivering Woman, FGD6*

When I am seeing my husband, I will have more faith. Faith. I will have faith. *Companion, IDI7, age 31*

When I was having a companion like my mother, I felt very easy for me to give birth. Because my mother was there, praying for me, And all the rest. Pampered me, encourage me so that I do not fear anything, let me put God first, you know? All the rest of it. I felt so comfortable when my mother was there. *Delivering Woman, FGD6*

I'd rather have someone, like my husband, to be with me. Because he will encourage me to have faith, encourage me, give me encouraging words until I give birth... talking to you, sometimes they will talk to you until that the pain, you will not feel the pain because they will talk to you. *Companion, IDI7, age 31*

Give me courage. Said, 'don't lose hope, you have to fight for your baby, it's your first time.' So, all those stuff made me to persevere. *Delivering Woman, FGD10*

The words made me feel good because from one talking to me, said, 'no, you have to do this for the baby, I know this is your first time. You need to push,' you know? So, when the time came for me to give birth, all the word that my sister-in-law was saying to me made me have the motivation and made me have strength to do what I have to do. *Delivering Woman, FGD10*

I was very happy when my sister was there, because she is the one motivates me to give birth. She is really the one who help me, because it was not easy for me. It was very difficult for me. But she encourage me so that I can give birth to my baby. *Delivering Woman, FGD10*

They were encouraging me to give birth. You know, 'this is your first child, you need this child, please, keep on the fight.' Encouraging... For example, when you want to push, you stop it. Then you will say, 'no, don't stop, keep the fighting, just push, you know, just push, one more time. Do like this, then so, that is all.' *Delivering Woman, FGD10*

When I'm by myself the pain was so painful, it's really painful. But when I have a companion – because they are helping me, they are giving courage for me to have courage, they pray, they give you what they need. *Delivering Woman, FGD6*

I don't know, the love, the warmth... the time I see my husband, I have more strength to deliver my baby. *Delivering Woman, IDI3, age 23*

Companion as proxy

One of the responsibilities I performed as a birth companion, I had to sign for the operation to commence. I have to sign the document that to say I am here, I consent, I am aware that this is it the procedure that is going to be carried out on my daughter for her to be able to deliver. *Companion, FGD4*

I do all the necessary things for her. Like, to fill out the documents, because if you want to discharge you have to fill out the documents that you are going. *Companion, IDI4, age 27*

If you die, during that process, the nurses, the doctors, it will be difficult for them to reach your people. To your family members. But with your companion by, it will be safe... If your companion is there, they will ask that person to sign on your behalf. But if there is no companion, so who will sign? If that person dies, who will take responsibility? So that is it. So, it is not good, not having the companion by. It is not good. *Delivering Woman, IDI6, age 29*

Not having someone during childbirth, it is not good, because in case they ask you about some information. They need some information about that person... Information, like the day we came in. They asked about my name. So, during that time, if I was unconscious, how would they know my name? So, my companion with me, supply that information, they need it. So if there is no companion, they won't have any information. *Delivering Woman, IDI6, age 29*

Choosing a companion

Male vs. female companions

Better treatment from husbands after witnessing birth

[Witnessing the birth] gives me more courage and patience to be with her. Because for a woman to give birth, it's not easy... So I am not thinking of doing anything stupid... On that day, my mind was telling what that what the other guys are doing – because let me say, after two, three children, more than, they leave their wife to go and marry another wife. I am quite sure if they normally allow them to be in the labor room, they would not do that. *Companion, IDI4, age 27*

When the men see how the pain is that we the women go through so that, where if they're in the house, they don't want to help the woman, the woman will try to tell them, remember, when I was screaming, in the labor ward for giving you a baby. It is better for them to see. *Healthcare Worker, FGD5*

The respect is more than before. Why? Because he was there when I am giving birth. He saw everything. So the respect is greater now than before. *Delivering Woman, IDI3, age 23*

I want more men to be in the birth companionship position, because in Africa, even all around the world, the way men do treat women sometimes, especially after having maybe like two, three babies with them and they now want another woman, of which they think, I need to move to another relationship, they will start to treat the woman with the three babies like trash. But if the

man is in there when the woman is trying to give birth, because of the suffering, the struggle the woman will be through before giving that baby, that man will have learned some lesson and see that it is really a good thing for me to love my wife whether good or bad time, because look at the pain she is going through for us to have this baby. So, yes, men will start to treat women right. *Companion, FGD9*

For example, I say like Sierra Leone, some men, they do not have respect for women. So when my husband is going to be there, he will know that women go through pain to give birth. For that he will have respect. For that he will not abuse you. *Companion, IDI7, age 31*

I remember what the doctor told me. He said, 'have you seen what your wife went through?' Said, 'promise me that you will be with her. You will protect her. You will handle her with care.' So I give my utmost promise. I am quite sure with that, even if a man is doing something stupid before, by seeing what the wife went through, that will change the man's concept. *Companion, IDI4, age 27*

Here, Africa, they don't have respect for women. If the woman is in labor, she will call, 'nurse! Call my husband for me. Let him come and see what I am going through.' *Healthcare Worker, FGD1*

When the husband come, we want them to see how women are struggle for labor. Because in Africa, they do not respect women. So when you see that women go through labor, how to give birth, when you go through with your wife, you have to encourage her, you pamper her, love her more than before. *Healthcare Worker, IDI2, age 40*

The husband, like when they come, they stand. Like, we the nurses, we do not send them much, because we want them to see. We don't want them to go out and come, no, we want them to see from the start to the finish. So that they will know. *Healthcare Worker, IDI2, age 40*

Empathy and emotional support

[My husband] will help me, because he know, when my husband will be at that particular time, he will know the pain that I am going through. So he will help me. But when my mother also will be there, my mother will help me, but my mother will think that, she was giving birth one, two, three times. But my husband now, he cannot think about that. He can only think that I have serious pain. He encourages me... My mother she will pity for me but not that much like my husband. *Companion, IDI7, age 31*

She has experienced it, I think she will know what you are going through. But like my husband will not know really the pain I am going through. But a female person can. If the person has already experienced that, she will have empathy on you. She will encourage you because she knows the pain. *Delivering Woman, FGD6*

A female person that went through labor, I know she will have that empathy. She feels the same way. *Delivering Woman, FGD6*

Some of them, they will not bother to give the affection... their only concern is the baby. *Healthcare Worker, FGD5*

Some husband, they only need the baby. They will be there for the baby. But not the mother. After they have seen the baby, they wouldn't want to know what's the status of the mother,

because it's the baby they're there for. But my sister, my sister can be there for all of us. *Delivering Woman, IDI12, age unknown*

My sister was there to direct me, and the nurses were there also to direct me, they be the same job. But when I saw my boyfriend there, that give me the zeal more to do something. Yeah. When I saw my boyfriend, I just wish he would just come and I said, here is the baby. *Delivering Woman, IDI9, age 23*

Increased bonding, unites the family

It united family more... it creates a bonding between the husband and the wife and the family also. There should be love. *Healthcare Worker, FGD1*

That time I'm there, it helped me to increase the love I have for my wife. Because I saw the actual pain my wife was going through. *Companion, FGD9*

The love is growing more, every day, every day love is growing. So you see, I'm thankful for that. *Delivering Woman, IDI3, age 23*

Some of them will have more love for their wives than before. *Healthcare Worker, FGD5*

Men are afraid

I think that for me, that is one of the major difference between a female and a male. Some men are afraid, they do have the fear a lot. *Companion, FGD4*

My first experience, my husband went there. When he saw me in pain, he was crying like a baby! I said, why you crying! He said, the pain, the pain! Because you are crying, the pain. So, it will make you cry too. So you need somebody that cannot make you see the tears. This way you will have courage. But if the person cry, what will we do? We cry. Because he did not have this experience. So he will just be crying. *Delivering Woman, FGD6*

I do not have the gut to stay and watch my wife put to bed. No, I am afraid. I do not like seeing the blood and I do not like seeing her crying, so I prefer to stay out. *Companion, FGD9*

My boyfriend know nothing about that, he was just inside there praying, oh lord, save my baby, save my wife. I want them both to be alive. That is all. He knows nothing about that, but he was just praying. *Delivering Woman, IDI9, age 23*

My husband is somebody that is quick to panic, you know? Afraid. So that is why I choose my brother's wife. Yes. Because the way I was behaving, so my husband gets nervous. *Delivering Woman, FGD10*

For my wife to be in short pain and crying, at that time, I was like, afraid or fearing for the life of my wife. *Companion, FGD9*

When I was coming for CS, my husband, he ran away... I ask my sister, 'where is my husband?' She says, 'he went outside.' I said, 'oh!' They will not be able to bear the pain. *Delivering Woman, FGD6*

He will start shedding tears. So that's the reason why you have to take somebody that will courage you, because maybe if your husband is there, he will be crying. *Delivering Woman, FGD10*

The husband reassure her, support her, but the husband, they won't be there for long to see their wife screaming. If there was a mother, a woman, there, a woman would be able to give her emotional support. She would be able to calm her down more than the husband. *Healthcare Worker, FGD5*

The husband, they don't, they are unable to see their wives crying. They do go out of the room. So that's why we need moreover we need women as birth companion. The husband will come and accompany, yes, but the women more to help us with emotional support. *Healthcare Worker, FGD5*

Most men, they don't understand. Most of them will come, but when they hear their wife's shouting, they will go. So, it's better to have the female as a birth companion than the male. *Healthcare Worker, FGD5*

Most of the male are afraid, they do not want to see! *Companion, FGD4*

But most times the male do refuse to be with the women as birth companion in the delivery room, I don't know why... most of them do not like to be with their wives. *Healthcare Worker, FGD5*

Women provide more expertise

Even if my husband would have opt to come, I would still choose my mother-in-law, for the reason being that, my mother-in-law is in a better position to help take care of the baby... My mother-in-law is more experienced in these kinds of issues. My husband will not be able to do what my mother-in-law can do. *Delivering Woman, IDI5, age 19*

My sister? Because she is a woman, she have experience birth pain, and she know better. So she was there to give me some experience. Do this, do that, with the help of your nurse. *Delivering Woman, IDI9, age 23*

I only call my aunty and my mom. Because my husband, he don't know anything. He just come and stand outside, doing nothing. So that I prefer my aunty because she has knowledge. *Delivering Woman, FGD3*

The females, moreover women who have gone through childbearing, they're experienced. When it comes, they will talk to the patients, their own experiences, input. Knowing their own childbearing, they will talk to the patient. So that's true, that will calm the patient down. *Healthcare Worker, FGD5*

The women more to help us with the emotional support. Because they will share their experiences that they go through with the patients, and the patients also will listen. *Healthcare Worker, FGD5*

Trust

What I want to say is, the person that you pick to take you there when it is time to give birth. So my sister, she is a sister, she is a mother to me. She is the only person that I believe I trust to take me to the hospital when the times comes to give birth. *Delivering Woman, FGD6*

I take care of her. She trust me so much, me too I trust her so much, because this is my blood. You can have so many brothers, but one will be among you who have love who have caring. Your problem is my problem. *Companion, IDI13, age unknown*

I would prefer my husband or my mother-in-law... those are the people I trust. *Delivering Woman, IDI5, age 19*

As for me, my sister is the only person I trust. She is like mother to me. Because my mother is not here. That's why I choose my sister. *Delivering Woman, FGD10*

Here in Africa, it all depends on trust. When you trust somebody, that will be the only person you will be allow that person to accompany you to the hospital, because giving birth is a critical way. It is a critical way in Africa. So not everybody you will trust, or you will allow to follow you. *Delivering Woman, FGD6*

It all depends if the companion, the birth companion and the patient's trust. Maybe she has a trust in them. It depends if the companion is able to make a trust in the patient. *Healthcare Worker, FGD5*

During the birth you will not allow any Jack and Jill to enter inside the place. Only the person you trust. *Delivering Woman, FGD10*

Trust. She's the one I trust. *Delivering Woman IDI12, age unknown*

I trust her. A lot. Because in bad, not everybody will trust them. Not everybody you will allow her to follow you. *Delivering Woman, FGD6*

Fear of witchcraft

If I am there with somebody that I don't trust, maybe I will be with the feeling that, the person will be doing something negative or traditionally to let me not deliver safely. So for that reason, I will not be in the labor ward with somebody I don't trust... maybe the person will be, like, making it difficult for me to deliver. *Delivering Woman, IDI12, age unknown*

Here we have what we call black magic. You can be trying to give birth, but since somebody is somewhere else doing some native procedures, you will not be able to give birth... We have had scenes of these cases. You push, push, push until you get tired and passed away... You can't trust everybody. *Delivering Woman, FGD6*

You don't know who is against you. Because, you know, in Africa, we have those native things. Some will be there, say, 'this one, I will make sure, say, I will kill them. I will be so hard on her. We do something devilish for you to have difficulty in terms of labor.' *Delivering Woman, IDI6, age 29*

Witchcraft might be involved or so many evil things, other evil things. Black magic might be, as well. They say that they have, stone. They will take stone and tie it there, and say that you are not going to give birth... They will tie it and say that you are not going to give birth, you will struggle there until you give up. So that's why, when you have pregnancy, you are going to the hospital, you want to take to the hospital when it is time to deliver, you can only call someone who you trust, if not your mother or your sister or a good friend of yours that you trust, you know that this person will not do anything to hurt you. *Delivering Woman, FGD6*

There are so many bad people out there. Maybe some, at times you don't even get quarrel with them, but they just hate you, natural hatred for you. They want your happiness, you know? Something like that... They will use the black magic. *Delivering Woman, FGD10*

When my stomach started aching, my sister is the only person that I told, and the child's father. I not tell anybody that my stomach is paining me... because that is our tradition. When you are in labor, you shouldn't tell anybody you don't trust, only person that you trust that you are in pain. *Delivering Woman, FGD10*

At times, when somebody is pregnant, for example we are going to the hospital, then you started telling everybody, 'I'm going to the hospital! I'm going to the hospital!' You don't know, because there are so many evil people out there that don't want you to give birth. So you know? So, they have their own ceremony that they will process for you when you came into the labor room, it will become difficult. *Delivering Woman, FGD10*

Our people usually tell us that, you don't have to escalate it, so that when the time comes, just move quietly and come to the hospital with someone you trust... People might do bad things, people might manipulate. So our people usually tell us that, don't tell anybody, be strong, move quietly, just tell your trusted person. *Delivering Woman, IDI6, age 29*

Trust by giving good advice

I believe her, that whatever she tells me to do, I will do it. *Delivering Woman, IDI12, age unknown*

She always give me good advice. If I want to go the wrong move she will advise me, this is not good for you. 'Do this, this is good, this is bad.' That's why I trust my sister. *Delivering Woman, FGD10*

That is why I said I trust her. Even when I want to do something wrong, she will say, 'no no no, this is not it, this is the right thing to do.' She give me advice... Whatever advice she render, when I follow that advice, it will come out positive. Yes, so I trust her. *Delivering Woman, IDI6, age 29*

Trust to be non-judgmental and confidential

The patient said that her mother is the one that will be birth companion. I ask the patient, 'why did you not choose your mother-in-law?' She said, 'no. Me and my mother-in-law are not so close. Tomorrow if me and her son have any problem, she will start to explain that when you are in labor, I am the one that come and sleep here, I am the one that come and did that for you.' She said she will explain, so that is why she prefer her own mother to stand by her. *Healthcare Worker, IDI2, age 40*

If my mother-in-law is there, I'm not worried about privacy. But if it had been my sister, I'm worried about privacy issue... anytime we will be having disagreement, or there will be quarrel with my sister, my sister would easily paste it on my face or rub it on my face that, 'the time you were giving birth you were crying like a baby, you were helpless, I was the one doing x y and z for you and your baby.' So I wouldn't want my sister to tell me all of these things. *Delivering Woman, IDI5, age 19*

If the person, if you don't trust the person and you have some skin disease around your private part, the person will start telling you in a quarrel, that 'the last time when you went for a delivery, I was the one that was there with you. You have a lot of skin disease, rabies, and the likes.' You understand? So they would say so, all sorts of things. *Delivering Woman, FGD8*

If somebody you don't trust is inside there, they will do that. He is the one to exaggerate the talk... When they say, you were giving birth, you saw crying, saw an issue like this, you have rashes. *Delivering Woman, FGD10*

Trust by sharing everything

A companion is the person that you trust, that you can share anything with. Yeah, so that it a companion. You should trust the person to share everything with the person. *Delivering Woman, FGD6*

Even her, when she want to deliver, she will call me on my phone... I too go there and pray for her, started massaging. I will do the same thing for her, and she will do the same thing for me. So she's the only person that I believe I trust, after my mother. *Delivering Woman, FGD6*

She's my second mom. She love me as her younger sister, I love her as my elder sister. So we grew up in love, in that bond. So I trust her with everything I am doing... She sees everything. I don't have any problem with that. I trust her with it. *Delivering Woman, IDI6, age 29*

Trust by support during pregnancy

There is trust between both of us, and that trust is everywhere. Because from pregnancy, whatever thing that is happening to her during pregnancy, she will explain to me. Then I will explain to her, what's the reason for that. And then after that, we will come to the clinic, and they will explain the same thing. So, by doing so, the trust is there already. So when we are here on that very day, whatever the thing the doctor says, I will just talk to her and let her do the thing. *Companion, IDI4, age 27*

Since the very first day she got pregnant, I was there... always buying her medicine, because she was in pregnant care. *Companion IDI4, age 27*

Since inception, my husband was there with me, like coming to the ANC, and all throughout the pregnancy, the three trimesters, my husband was there with me. So I believe my husband could be in the best position to come with me to the hospital. *Delivering Woman, FGD8*

I trust my aunty so much. During clinic hour, she will be there. When I go to clinic, she will make sure I see a doctor. And from clinic, from when I am coming to clinic she is there, she will take m card and go everywhere for them to touch my belly, to know how the baby is doing. So she is doing so many things really in pregnancy and birth. *Delivering Woman, FGD6*

My aunty, since I started giving birth, she is the only one that is accompanying me to this hospital. I trust her. I believe her. *Delivering Woman, FGD3*

The love from one of my pregnancy, my older sister, she was there for me so much. Pampering me, 'do you want this? Do you like this?' At times I would talk one of my sister. If need something I don't like, I would talk on her. So you know? She was there for me and loved me so much. So you see? That's trust. *Delivering Woman, IDI3, age 23*

Implementing companionship

Reasons for previously not allowing companionship

Concerns about privacy

I thought that it would not be possible. Based on the patient's privacy, some relative come in, so I was thinking that it would not be possible for it to work. *Healthcare Worker, FGD2*

Some relatives are coming, maybe the patient is not the near relative. Maybe in labor, help them to come. Accompany them in the hospital. So for the birth companion to see the patient's nakedness while giving birth and go out there, some would not know certain things, go and explain everything. *Healthcare Worker, FGD2*

For some, when they come, don't pay attention where they come. They start to invade another patient. Yes. They want to know, what happened to this patient, what happened to that. They leave their own patient. *Healthcare Worker, IDI2, age 40*

The only thing that makes me – because, the space is a problem in the labor ward... we have to do another thing for the spacing. For the birth companion. Because they don't have seats in the labor ward, they can only stand. *Healthcare Worker, IDI2, age 40*

I was thinking it wouldn't be possible because of the space. You have much space, you have special room for each patient, they just single, so this room divided by curtain. So I was thinking it wouldn't be possible at first, when the information came in. *Healthcare Worker, FGD2*

When they are coming, they come like, five or six people. They say these are my relatives. So we say, outside, only the patient will stay in ward. *Healthcare Worker, IDI2, age 40*

The spacing, that is a major issue... Some people say, I want my husband, or some people, their husband will just be so arrogant. They can't wait outside, they want to stay inside with their wife. But would you allow another man to see another's wife naked? *Healthcare Worker, FGD1*

Privacy. For me, that was my big thing I think that will dread them before. Because some of them, when they saw how the women push, or they saw how the woman's private parts is. Maybe, me and you are friends or neighbors. Tomorrow if we got misunderstanding, we have seen everything that in me here, maybe you will stand outside and explain about me. Talk about me. Yes. You have come here, you have seen everything about me. My private parts, you know everything. Maybe tomorrow me and you are home, we have misunderstanding. You started explain, 'when you go give birth, I am the only person with you! You have so-and-so problem! This is that, this is that.' So that was our fear, I think. *Healthcare Worker, IDI2, age 40*

Threat of theft or scamming

Sometimes, these people that are coming are not the relative of the particular clients. Neighbors, yeah, some are not related. We have seen cases... this lady was talking to the client admitted to the ward 6. We thought she was a relative to the patient. She steal the patients' phones! *Healthcare Worker, FGD2*

Some of these patients, they are two-sided person, some of them pretend. 'Do you know this person?' 'Yeah, I know her.' But time goes by, they don't even know the person, just because they are in pain need somebody to be with them more than the nurses. They will pretend as if they know the one that's accompanying them. Maybe the labor starts accidentally, when the patient is working, outside. So the one that accompanies her, she will pretend as if she knows this person. *Healthcare Worker, FGD2*

Lack of space and resources

When the patient is coming, he or she will come with 6 or 7 people. But you ask the patient, 'who do you want to let him or be with you?' Then she is the one that will pick the one that want to be with you. So I said that, 'oh, so this is the birth companion.' *Healthcare Worker, IDI2, age 40*

Overcrowding. Some will come with this and this relative. The grandmother, the mother, the husband, the sister. Overcrowding the labor ward... before we said no. We asked them to go and stay outside. When we need something, we call one relative to give us. But now, with the introduction of birth companionship, we ask one relative, take their name. But with the consent of the patient. *Healthcare Worker, IDI8, age 50*

Five, six people accompanying only a single patient. Overcrowding! It's nice if at least one, at most two, people accompany patients, it's simple. But with five, six people accompanying the patient, with the small environment we have, so that's why it is difficult. *Healthcare Worker, FGD2*

One patient come in with five relatives, so we ask them out, all of them, unless at times we need one of them, we call one to come in and give us the information. But we are not practicing this birth companionship perfectly before March. Then spacing in the labor ward also was another problem for us, because spacing was very small for us. So that's why we are not practicing it perfectly before March. *Healthcare Worker, FGD1*

The set-up of the labor ward was not conducive for having birth companion... because of space. We are having a small space, where we don't have a place for the birth companion to sit, either to stand or to witness the delivery. *Healthcare Worker, FGD5*

It is a lack of resources. There was not adequate resources... like the extra scrubs, extra footwear, like that. *Healthcare Worker, FGD1*

Arguments with relatives

For some of them birth companion, they do not come with one, they come with three, four relatives. Some of them wait outside, they are standing, when the space was so small. They stand outside, waiting. So some of them, they quarrel. They want to be inside. They quarrel with us. Because we don't allow them to get inside the ward. They say all sorts of talk against us. *Healthcare Worker, FGD5*

When they get home, they talk all sorts of news against us. They said the PCMH nurse did not allow us to enter into the hospital, into the wards, they are changing babies, that's why they don't allow us. They are changing sex, that's why, the propaganda the sister is saying. *Healthcare Worker, FGD5*

For example, when they come to the labor ward, they see different babies in the warmer, in the pots, they see different babies. They won't understand that those babies are identified. They have different wrappers, their names, their sex, their information are in their hands or inside their wrappers. They won't understand. They think that we will mistake another baby for their own baby. *Healthcare Worker, FGD5*

Educating the companion

Hard for us, but we just need to do it. Because they need to be there, there should be birth companion, that we know. So we just try to talk to them, there's more talking for us the nurses – more talking, education, we educate them every day. You have to be doing it, it's on a daily basis. Every patient comes in, you need to talk to them. The same message you need to pass to them every day. *Healthcare Worker, FGD2*

The birth companions is not there to do the job of the nurse. I educate her, 'do not do the job of your nurse.' We are only there to monitor your patient or to help her, because some patients are uncooperative. *Healthcare Worker, IDI1, age 36*

All the birth companions, I told them. Because when you are in this room, you must hear another patient over there, she is screaming, torturing, saying all kinds of words, abusive language. She is in pain. So at times we told them, 'when you are here, whatever is in here, inside here, let it remain here. Please don't go outside and explain that this woman is in labor, this is the word that she said.' So we told them. We explain to them, we teach them about that. *Healthcare Worker, IDI2, age 40*

I told them, 'you have come. This one has choose you that you supposed to be their birth companion. When you go outside, please' – I talk to them – 'what you see here remain here. Don't you go and explain to others that when she is giving birth, so-so-so-so-so, she has that, she has that. I say no, I do not want that. What you see here remains here. She's your friend. You have to keep her secret. Don't go and explain to other people.' *Healthcare Worker, IDI2, age 40*

When you receive the pregnant woman with the birth companion, you have to educate the birth companion what to do and what not to do... So you the nurse should educate the birth companion. *Healthcare Worker, FGD5*

You tell them, 'are you the one that will be with the patient in the hospital, the birth companionship? And since you are here, the only thing that you should do is talk to the patient, because most of them don't respond to pain, they just get fear. So you the birth companion are here, you will try to alleve [alleviate] the fear, talk to her, to be calm until the time is for the baby to come.' So that's the education we give them. *Healthcare Worker, FGD5*

Now after we have done this option [of birth companionship], we are educating them... we talk to them, that they should be near to their relatives. Or the husband, the husband will be with his wife to deliver. Because he is the husband of that one, so he needs her, to take care of that one, the one she is in labor, he has to be with her, there for her. So he will be there to do the work. Some of them accept. *Healthcare Worker, FGD1*

We tell them, 'if there's an IV done, no need to touch anything, just call our attention... You should not do anything. Only for the woman, just give her emotional therapy, that's all.' *Healthcare Worker, FGD1*

We tell them, 'since you are the one that came with this woman, please, for anything you want to do, we just need your moral support, your reassurance. We don't need you to do anything else. If you want to give this pregnant woman food, or anything else, you ask us.' *Healthcare Worker, FGD5*

Monitoring the companion

We don't want to leave them there alone. *Healthcare Worker, FGD2*

Some birth companions, you have to monitor them. And if not they will be doing extraordinary thing. *Healthcare Worker, FGD2*

The advantages better than the disadvantages. But you know when they are in there, you don't leave them there, you should monitor them... because they will do what they feel like doing. Which is not good for us and the patients. That's why when they are there, we don't leave them all by themselves. We have to go monitor, every ten minutes, every twenty, thirty minutes. *Healthcare Worker, FGD2*

You know these traditional things, they will smuggle them. But when they come for my own I will search them properly, remove the things, the birth companion I will search them thoroughly to see if they have any of those things near the patient. *Healthcare Worker, FGD5*

We need to monitor them. We advise them, with your patient, your own space and your own area. *Healthcare Worker, FGD2*

Knowledge of importance of companionship

Seniors were not implementing

When we started doing delivery, that's what we met our seniors doing. They don't allow relatives to be in the labor ward. *Healthcare Worker, FGD5*

The idea was there, because when we go to our midwifery schools, the idea was there, but like in our setting we don't know if the people really appreciate that or needed that. *Healthcare Worker, FGD1*

Lack of knowledge

Before March, there was nothing like that, really... Maybe because of the spacing or because we are not considering that, that is it important... We did not know the importance before that time. *Healthcare Worker, FGD1*

Before, we drop [the companions] out, because we don't know their importance. We thought that it is not necessary when they came. *Healthcare Worker, IDI2, age 40*

I did not have a clue. Only outside the workshop, I know exactly what is meant by birth companionship. *Healthcare Worker, FGD2*

We would not have the knowledge that patient is supposed to have birth companion. *Healthcare Worker, IDI2, age 40*

You ask the patient, the person that comes and stay with the patient from labor till to the end. That is the one that birth companion. But before, we did not know that. When they came, we drove them out... we did not know that, it is very important for us. It less our work. *Healthcare Worker, IDI2, age 40*

Workshop, institutionalization means easier implementation

Because everybody has gone through the training, they know that you should exist, you understand... Before if you say, this patient needs a birth companion, people will start, 'why are you like that!' Asking questions. But because everybody is aware now, they have come to the workshop, the training, so everybody is aware, so everybody makes effort so that the birth companionship can go through. *Healthcare Worker, FGD1*

You bringing this program to us, it makes it easier, because we tell the theatre sister or other, this patient needs a birth companion so you should allow. So it is easier now than before... Because you guys came with this program everybody knows that it is OK for somebody to go into the theatre so long as she has changed and the nurse stay with you. *Healthcare Worker, FGD1*

Satisfaction with companionship

The good outweighs the bad. Maybe there is a hundred good, there is just one bad. *Healthcare Worker, FGD1*

It's a very good initiative for us as the healthcare workers. *Healthcare Worker, IDI2, age 40*

The birth companions, it is very very good, and we are happy that we have it now at PCMH. I hope it will extend in the provinces too, so that they too will learn more about this birth companionship. It is very important. *Healthcare Worker, IDI2, age 40*

Advantages for nurses

Reduced workload

When you have birth companion, your work will go easier for you. *Healthcare Worker, IDI2, age 40*

It reduced the workload. The workload in the labor ward. It reduced a lot for us. You see some patients, they will just be screaming, 'I want to do this, I want to do that.' You will check, maybe the dilatation is still four or something. Maybe she has something, maybe for days she hasn't gone to the toilet, and in labor she will always feel like she wants to go to the toilet, so they're always around to help. Giving them a stool to sit and so they can defecate. Other things, maybe when they want to throw up, maybe the nurse will not be there to offer the vomit bowl. So they will help in giving the bowl to throw up so it doesn't splash on the other side. It really reduced the load of the nurses. *Healthcare Worker, FGD1*

Everything is easier for us. *Healthcare Worker, FGD5*

The workload is easier. Easier in some cases, because just as I was saying, maybe the patient is having a thing. She said, 'hey, my back is aching me.' The relatives at times will assist. *Healthcare Worker, IDI8, age 50*

As health worker, when you say the woman is in labor, I have to do everything for her when there is no birth companion. If she wants water, she told me, 'nurse, I want to drink water. Nurse, I forgot my *lappa* over there. Go and buy this for me.' But if birth companion is there, she will go for water, she will go and buy *lappa* for her, even if they forgot to buy pad, she will go out and buy pad. *Healthcare Worker, IDI2, age 40*

It is easier for us. The job will go easier for us... because when we have 3, 4 patients in labor. All of them have their birth companion. I the health worker will sit here now and write my notes, if I have done all my investigation. I will sit and write my notes. The birth companion will stand at the patient, he will be know, if something will want to go wrong, she will call me, nurse! Something is going on. So I will come and see. But before when there was no birth companion it would take us a long time for write our notes. Because we have to do everything. *Healthcare Worker, IDI2, age 40*

Before when there was no birth companion, when I was working in the ward, like in the morning I have five or six baby to take them for BCG. I will hold one here, one here, two... I came again, I took more. So I walk more then. But from now, where there is birth companion, this one hold her baby, her daughter's baby. This one hold this one. I just there for supervise them. Let's go... We do not walk more. So that is very good for me. *Healthcare Worker, IDI2, age 40*

When they are around, it really eases our stress. Because for us, the nurses and midwives, we want the patient to come, suffer labor, and at the end of the day, the woman and child is alive. But when the relative is around, they will run around and get the things they need for emergency cases. Then, the patient's life has been saved, and the baby as well. *Healthcare Worker, FGD2*

Companions help physically during labor

For some of them, while giving birth, they will fight! The relative will help to hold their arms... And you know, by closing the legs, it will put suppress on the baby, and it will suffocate the baby. So the relative, the companion will help us. Hold their legs, hold their arms. *Healthcare Worker, FGD2*

My sister was there, holding me firmly... My sister was there to hold my leg, put it at one place and concentrate. *Delivering Woman, FGD8*

Some patient during the second stage will try to close their legs, their thighs. The companion will ask to assist you, hey, just to open their legs, for the baby to be delivered. Some will assist you by holding the thigh. For easy access, you see. *Healthcare Worker, IDI8, age 50*

Companions can fetch medical supplies

The hospital, we have a lot of constraints. If the birth companion is there, we will be able to send the birth companion to go and get this, go and get that. But if the patient is alone, you have to be running around to find ways to get what she needs for her treatment. *Healthcare Worker, FGD1*

We knew if she was going to the operating theatre so we have to get all the premedications that you are prescribed... we had the ones that were prescribed, and the ones that were available we were giving. Like, IV fluid was given, because by then, there was no urine bag and catheter, so she was catheterized. *Companion, FGD7*

We saw some medical people at the entrance. They told us to get some medicine for her. To buy some drip. I give them some money, they take my money and they buy some drugs. *Companion, FGD4*

They ask for something like the catheter... It's the responsibility of us to provide it, that is if the hospital run short of it. Gloves as well. If the hospital run short of gloves, we are the one that should provide them. *Companion, FGD9*

At times with supplies, at times supplies are scarce... Maybe the sutures are finished... We have a lot of patient inflow. So, due to that, the sutures, things that they need, supplies, even gloves, supplies will finish, because you have a lot of inflow of patients. But, a nurse will not go about running to the pharmacies to buy those things. But when the relative is around, it eases the tension for us. Because the relatives will go, they buy the things, they go and the patient is saved. *Healthcare Worker, FGD2*

Relief from providing emotional support

A birth companion lessens the workload on nurses. Maybe if I'm there in the delivery and the birth companion will be there to comfort the patient on the bed, rubbing her head, massaging the back, and the nurses can do her job. So it's much better that the birth companion is there. You can keep on performing the delivery and she says, 'nurse, my back is aching!' And then I will tell the birth companion, 'can you please help me to massage her back?' So it's much easier. *Healthcare Worker, IDI1, age 36*

It makes it easier, because of these patients, who are having difficulty... when their birth companion is there, they have confidence in their birth companion. The birth companion will be there explaining to her what to do and what not to do. Then the work is easier for us. *Healthcare Worker, FGD5*

At times, because we the midwives at times we'll be working here, working there. But with the birth companionship, with the relatives that the patient has chosen, we also help the patient by reassuring her. This eases the pain... We do reassure the patient, but at times with the workload, we say, wait here, I'm going to do my partograph, I'm going to do my other work. So with that, we will not be with the patient totally. But with the relative, because they are so stressed, they will be by that patient, you see. So that also will help. *Healthcare Worker, IDI8, age 50*

Communication

Language barriers and translation

Another thing is they also help in case of language... Some come, they will just be like, I don't speak English, I don't speak Krio. So just the native language... So for them to communicate it's not that easy. So most times they come with either their neighbor who will always be with them or maybe with someone that is related to them. Or maybe their co-wife or something will also be with them. They will now be the one interpreting to the nurse. *Healthcare Worker, FGD1*

Some will not understand the Krio language. The language barrier. So they only come with birth companion to explain everything. *Healthcare Worker, FGD5*

That time, the baby is Temne, that tribe. She will not even understand Krio. So the birth companion explain to her in Temne. *Healthcare Worker, IDI2, age 40*

Like the delivery I saw this Thursday, because of language barrier, that made me go back, I love this birth companion! For language barriers... Some of them do not understand Krio, neither English... So when this birth companion came into the facility, we are so happy for that. So when the patient came, she did not hear any Krio or English. We called the birth companion, she interpret the word for us so that we can understand what she is talking. *Healthcare Worker, IDI2, age 40*

Companions provide reliable information

When the patients are coming to the labor, they need somebody to accompany them and then get information from some of these birth companions... Sometimes we ask the patient, how many times have you delivered? And some of them will say, two times, maybe it's five times. So they don't give the correct information. And some of their birth companions give us the correct information about them. *Healthcare Worker, FGD5*

They discuss about their personal information before coming to the facilities. So when they came, some of them are in severe pain, so they do not – when you ask them about how many times, the parity or gravida, they will not say the right word. But because of their previous conversation to their birth companion they will ask them, they will give us the correct one. *Healthcare Worker, FGD5*

If the patient comes in like those our referral cases. Most will be stressful, because they have been in the clinic or health center for some time, let me say one or two days. And she's very tired. Even to speak, she will not be able to speak. We ask the companion to give you all the details. Some will even say they're a referral, 'we have been there for two days...' She will tell us everything, some companion, yes she will tell us everything. So with that information, we taken them down for further. We take the information down. *Healthcare Worker, IDI8, age 50*

Not having someone during childbirth, it is not good, because in case they ask you about some information. They need some information about that person... Information, like the day we came in. They asked about my name. So during that time, if I was unconscious, how would they know my name? So my companion with me, supply that information, they need it. So if there is no companion, they won't have any information. *Delivering Woman, IDI6, age 29*

Maybe she bleeds at home before coming. You see? Some will assist us. Because some will not give us the correct information. Because of this, some will travel with bikes and because of the road network, some patients will have a rupture, they will come to this hospital, they will not explain to us exactly what went wrong. But with some relatives, they say, 'eh, no, nurse, this and this happened. She was travelling with a bike so she started bleeding.' So that will also help us. It really helps us with some cases, they will explain exactly. *Healthcare Worker, IDI8, age 50*

When we reach there, the first thing they ask us, they want to see the document where the doctor signed... They ask for those things, and they ask if we've signed the consent form. And we said, yes. And they try to know if she has been catheterized. And we said she has been catheterized at ward 6. *Companion, FGD7*

Because they are undergoing pain, so they cannot give us the right information. Some of them if the pain is not severe, they could give us the right one. But if they don't give us, we ask their birth companion to give us the right one. *Healthcare Worker, FGD5*

One case I delivered this morning. She's 17 years old. She came in with her sister... She's HIV-positive. So after, before delivering, the birth companion told me that the counsellor working... said after delivering the baby, she will give you the baby that syrup. The birth companion told me that. So I was shocked! *Healthcare Worker, FGD5*

Companion as witness

Protect nurse from false claims of abuse

You have to be careful when you are talking to these patients. The relative should be there to see what is happening. *Healthcare Worker, FGD1*

Some of them give the wrong information... When they are alone... if you have done the deliver then everything is OK. Unfortunately there is a small mistake in there, they will also say all sorts of words against the nurses. *Healthcare Worker, FGD5*

We tell them, it is better you are here, because you see what we are doing. *Healthcare Worker, FGD1*

If there is no relative in the room, you can say anything about the nurse. But when the relative is in there, the relative will be the witness, so the nurse will be free to do her work. That is one advantage for nurses. *Healthcare Worker, FGD1*

When the birth companion is with the patient, she can see everything that is happening in the room, so, there will be no allegation against the nurse. So the nurse will be free to do her work and relax. *Healthcare Worker, FGD1*

When a patient is alone she will go and say a negative thing about the delivers. But if the birth companion is there, he will say no, this is what happened. *Healthcare Worker, FGD5*

Protect nurse from false claims of baby theft

When there is a case from another facility and they have said it was a multiple pregnancy, one, we ask, even if you want to go, when your patient is going to second stage, we don't allow you to go outside. Not only the patient should know, but the relative should stand there, to know the sex of the baby. Then if it is multiple, because at times the scans will tell you that it's multiple and it's single. So we do ask the relative to be there, because we don't want problems to occur... Because some patient will tell you, hey, you showed me but I was not seeing properly. Some mothers say yes, after I have delivered show showed me the baby but I was sleeping. But with the birth companionship, it is very necessary. *Healthcare Worker, IDI8, age 50*

You know, sometimes we hear a lot, in terms of hospitals. Yes. All over, government hospitals. They said they were, in terms of theft, children missing, those kinds of stuffs. But with your companion by, maybe you are safe, even if anything or anyone want to approach, you are safe. *Delivering Woman, IDI6, age 29*

Some relatives also believe, PCMH, generally, we steal babies... So now there is birth companionship, there is no stealing. There is no accusation, because the birth companion is there until the woman delivers. *Healthcare Worker, FGD2*

We the nurses are having so much blame that we don't care for our patients, that we do exchange babies. But since they're there now with the patients until the patients deliver, it is an advantage for us. Because when they are there they swill see everything that happens. And give correct information outside. *Healthcare Worker, FGD5*

Most of them go with that idea, that they will exchange their baby, will stole their baby. But when their birth companion, come, as soon as they have delivered, they ask their mother or their birth companion, 'mummy, watch my baby! What sex?' But in minutes when the baby has come I will show them what sex the baby she delivered. She told us male or female. She turn to her birth companion, 'go and watch my baby, watch it.' So they also accept this birth companion. They are happy about it. *Healthcare Worker, IDI2, age 40*

When I put to bed, I ask my aunty, I said, 'which of the sex did I put to bed?' She said, 'female.' *Delivering Woman, FGD3*

Some will reject when you tell them that this baby is IUFD. Some will say, 'no, my *pickin'*, no, my baby still alive.' But we still tell the companion. So she will not accuse you that you steal the live baby and give her the dead baby. With companion, It has really advantages. So many advantages that will help the nurses. *Healthcare Worker, IDI8, age 50*

When we are having birth companionship, it's very nice for us. Because before, some of this birth companionship, especially the husbands – they are eager for a male child. Even they don't do scan, they want a male child. But when they accompany their wives to the hospital and the wife deliver a female child, oh, that was the time they started grumbling, talking about they are exchanging the sex of the baby. I was expecting a male child. So that was our constrain before. *Healthcare Worker, FGD5*

After delivering the baby you show the mother what did you give birth to, and tell the baby, put the baby on top of the mother's chest, and we call the birth companion. So, the pregnant woman has given birth. What has she given birth to? And she told us. So birth companion saved us. *Healthcare Worker, FGD5*

I was in the provinces, but sometimes we cannot hear good news about this hospital. Like they said, some people say they carry their baby, some people say they exchange, so sometimes for that reason the nurse can say, we cannot allow everyone to be where the particular woman will give birth to her baby. *Companion, IDI7, age 31*

So the birth companionship is very important, because then they're around they've seen, they know what happened, so it even releases stress for we the nurses and saves us from a lot of problems that were going – that at PCMH nurses steal children, steal babies. *Healthcare Worker, FGD2*

Also for our safety, that is most important. Because now for these law issues here in Sierra Leone. Because these cases, like what I said, that the patients who say, 'when they went to the ultrasound scan, I had a girl. But now, it's a boy. They have changed my baby.' If the birth companion is not there, what will we do? So it is very important for our own safety. *Healthcare Worker, FGD1*

Whenever there is a caesarean section in the theatre, we make sure that from labor ward, the nurse go down the relative to collect the baby also while the nurses is inside the theatre, the relative will stay by the nurse, the nurse that is going to receive the baby, so that the relative will see what the sex of the baby. *Healthcare Worker, FGD1*

We tell them, it is better you are here, because you see what we are doing. Because some people when they do their scan, they will say it's a girl. Then when the baby is here, it's a boy. So we have those controversies. We tell them, that is another reason why you are here... You should stay here and know that we are not changing your baby. *Healthcare Worker, FGD1*

Easier to deal with patients

Companions know the patient best

The woman was in labor, the head had come out but the baby was not coming out. So the relative took a *lappa* and gave the patient, the birth companion, she gave the patient, and they are going like this [pulling back and forth]. The woman started going. The process, once they were going, the woman delivered peacefully. The only work for the midwives that we are standing there was just to receive the baby. *Healthcare Worker, FGD2*

My sister was in pain. The nurses tried their best... but my sister was too lazy, so they call on me. Said '[name], come on! Your sister, they make quick quick for born, the *pickin'* don't go born. If you not come, your sister will lost her baby. So come and help her.' So I went. I talked to her, prayed for her, but she was too lazy. So, I implemented a style for her to give birth. The style that, I took a *lappa* and give it to her for her to draw me and then I too draw her. So in that process, she started drawing me. Then the baby started to come out. But she let go again the *lappa.* Then I said to her, 'please, please I beg for the sake of god. Hold the *lappa* and draw me.' Then she hold the *lappa* again. She was drawing me, I too was drawing her. I said, 'draw with all your might!' Then she started drawing me. I draw her. Then the baby shoot out. The baby come out. So that was how it happened, because I was there from the beginning to the end. *Companion, FGD11*

One of the usefulness of this birth companionship is they will explain to us the traditional side of the – maybe you will say, nurse, just give her this packet of water. As soon as the patient drink the water you will see. *Healthcare Worker, FGD2*

The relatives, because they have been with the patient... they know the patient. They know what to say to the patient so that she will become calm and listen to the nurse. *Healthcare Worker, FGD1*

Patients become more cooperative

Even with patients that are not cooperative, maybe don't be making noise, baby coming, they will be misbehaving. But when they have their relative around them, they will talk to them, they will calm them down. So it's important. *Healthcare Worker, FGD2*

I even told the nurse, 'remove the drip, I don't like it...' My sister will encourage me to take the drip. *Delivering Woman, FGD10*

They will assist us by talking. Encourage the patient to push during the second stage of labor. They will talk to them, 'hey, the nurse is saying you have to push during this second stage, or the

labor must be delayed. Push, push.' Yeah, some will listen to their companions. *Healthcare Worker, IDI8, age 50*

When the baby want to come, she will clamp her foot like this. The birth companions help me to talk to her, to open her leg, so that the baby will come out. When the baby want to come out she lock her leg, and the birth companion help me to talk to her. *Healthcare Worker, IDI2, age 40*

They will make the patient calm, they will be able to communicate with the patient. Even if the patient is stubborn, the birth companion will be able to communicate with them, give them reassurance, explaining to the patient. *Healthcare Worker, FGD5*

They also make communication easy with the patient... We encourage them, talk to them. Tell them, 'you see, I give birth to you, you also have to help' – if their mother is there. They tell them that this is the way we too give birth to you. So you too have to do your own and give birth to this baby. They encourage them, talk to them. *Healthcare Worker, IDI2, age 40*

Especially the primigravidas, at time when you want to do the vaginal examination, they are very uncooperative. So we ask the relatives to come and talk to them, that the baby is going to come out from the vagina. So they do talk to them. So with that, at times, they cooperate with their relatives. *Healthcare Worker, IDI8, age 50*

As a nurse, when the birth companion is with the patient, the patient will listen to the birth companion. She listens so much. But some patient, they fear nurses. For example, if you want to give them IV. They are being afraid from needle. So the birth companion will be able to hold the patient for us, to calm them down, 'please, be calm, just to help you to deliver, please.' *Healthcare Worker, FGD5*

When the nurses are also there with me, if the nurses want me to make some move, my sister will interpret, the nurses want you to do this, they want you to do that... my sister was asking me to push, it was time for delivering the baby, so she was like, encouraging me to push the baby. Delivering Woman, IDI12, age unknown

Some of these patients, when it comes time to do their vaginal examination, they will refuse. But if the companion is in there, she will talk to the patient, please, relax, let them do their work. So that will give us space to do our work. *Healthcare Worker, FGD1*

There was a woman in labor, she was not too cooperative, but fortunately the husband came around... the husband was fanning her and rubbing her head like this. So because of this, she lay down quietly, she cooperated and she delivered. *Healthcare Worker, FGD2*

Even when I said my feet are getting weak, she said, 'no, don't say so. Because the nurses say that, lie down this way.' I said 'I'm tired, I wanted to lie down on my tummy. I want to lie down the way I want, I am tired.' [The companion] said, 'no! Just continue to sit up.' *Delivering Woman, FGD6*

The companion reassures... emotional therapy, talk to her, to cooperate, whatever the nurse want her to do let her do it... So she will cooperate with the nurse, whatever the nurse want her to do let her do it. *Healthcare Worker, FGD1*

Most patients, when the come they have this fear, they are stubborn. Most of them they'll refuse to lie on the bed, they'll prefer to lie on the ground. So the birth companion will now try to talk

to the patient. 'Please, I am here with you, to help you with this deliver. I want to hear the cry of your baby. Please, cooperate with the nurses...' So she'll be able to talk to the patient, to calm the patient. *Healthcare Worker, FGD5*

They will assist you by talking to the patient so that patient will cooperate... We are having one case... I asked the patient if I should call the mother. The mother was in. With the mother, she was very cooperative. So the mother was rubbing the head. When she was trying to close the thighs, the legs, so the mother asks her to please open, she will deliver quickly, by opening the leg... She listened to her mother. She was very cooperative when her mother was there. *Healthcare Worker, IDI8, age 50*

When her husband is there, is present, they become more comfortable. Talking to her, rubbing her back. Make cooperative. *Healthcare Worker, FGD5*

It makes it easier to communicate with the patients. Because some patients, if you are telling her, try, push this baby... we will call the birth companion, please, come and talk to your patient. Then, she will listen to the birth companion and cooperate. *Healthcare Worker, FGD5*

So the work is easier for us with the birth companion around. Like those teenage girls, they bring their mother. She will not be taking anything you are saying. But when the mother is there, talking to her, reassuring her, they will be more than you the nurse. They will just be doing like that. But as soon as the mother comes, talk to her like that, then she will. *Healthcare Worker, FGD1*

Maybe so if you tell the patient, please climb on the bed and lie down, she will say, 'no. I'm used to give birth on the floor.' She says, 'I'll lie down on the floor and give birth there.' Once, when the birth companion is in, I say to her, can you please climb on that bed, encourage her, and the nurse is also there to encourage her to cooperate and climb on that bed to give birth safely. *Healthcare Worker, IDI1, age 36*

So I went and called her partner. Well, he came immediately and she put him to bed, she was happy her husband was there. *Healthcare Worker, IDI1, age 36*

My sister, she was there, like, lie down like this, the baby will come fast, don't push when they don't tell you to push. Yeah she was guiding me... When I want to come down from the bed, she say, 'don't come down. Lie down. Don't give us trouble.' *Delivering Woman, IDI9, age 23*

You see those women that are covered... when they come in the labor they don't want to remove the hijab... but if the birth companion is there, she will reassure, that no, you should remove, after which you will put it on again. But allow the nurse to see that. So it is much more easier now. *Healthcare Worker, FGD1*

While the woman is in pain, the nurses could not say anything to them. We are the rightful person to talk to her at that time. So whatever the nurses tell them, we will just do the needful for the procedure to stay true. *Companion, FGD9*
When they, the nurses, were asking the patient to push, the lady was not understand, how do I push? I was the one who said, 'just pretend you want to use the ladies', like you want to poo, you want to do number too.' Then she said OK, she started pushing until the start experiencing the baby. So I was the one who gave her an overview of how to push. *Companion, FGD9*

I was here consoling her. You know they normally ask her to push, push, so I was there talking to her, to do as the doctor was telling her to do. Because she was finding it very difficult, because she asked the doctor to stop putting hands on her. And I said, it will not possible. They just have to put hands on you... She was very angry because she said they shouldn't put hands on her. And I said, this is delivery, they just have to... So I think, that's the main advantage of the man being there for the wife. Because, when you say something, the wife will listen. *Companion, IDI4, age 27*

Disadvantages for nurses

Inappropriate behavior

Encourage or engage in physical and emotional abuse

They will come with the pregnant girl, very annoyed. 'I sent her to school, and she didn't go to school. She said she want to get pregnant.' She will start to be talking, and I will say 'no, this is not the time for this. You should have done this before, not now when the woman is in pain.' *Healthcare Worker, FGD5*

Some of them, they do not know how to talk to nurses. They want to be there aggressively. *Healthcare Worker, IDI2, age 40*

Some birth companions are very uncooperative! They want to show you how to do your job. *Healthcare Worker, IDI1, age 36*

Some of them, when they come, they want to tell you, do this do this to that. The birth companion. *Healthcare Worker, IDI2, age 40*

Some relatives they are very... they will try to dictate to the nurses. *Healthcare Worker, IDI8, age 50*

Some of the birth companions even ask us to flog the patients... Yes the companion, when they are making stubborn, and the birth companion says, 'hit the patient so that you can deliver quickly. Even I was, I will flog her. Nurse, don't joke with her. Flog her! She will lie down and deliver. If you don't flog her, she will not be cooperative.' *Healthcare Worker, FGD5*

Companion makes the patient less cooperative

Most of the time, maybe the patient will accept their own view more than the midwives or nurses. Because they will know the birth companion, the relatives, maybe their sisters. Especially the females, or the big sisters. Especially those TBA. Traditional birth attendants. Yes, they will listen to their word more than we the nurses. *Healthcare Worker, FGD2*

In one case I delivered, the birth companion, we have both of them, the husband and the husband's sister. But when the husband's sister is around, the pregnant woman will listen to her. But as soon as the husband comes in, she will drown and drive the husband out... She will get annoyed, very furious... Some of them don't listen to their birth companion. *Healthcare Worker, FGD5*

Another disadvantage we are having is some of these patients are very stubborn when their relatives are there... We say, 'we want you to cooperate now, push, turn to your left.' But because the companion is there, they will refuse. Sometimes. *Healthcare Worker, FGD2*

Interference

Interference with medical processes

Some of them, when they come, they want to tell you, 'do this, do this, do that.' The birth companion. *Healthcare Worker, IDI2, age 40*

The relatives usually give problems to we the nurses working, sometimes... They want to play the role as a midwife, as a nurse. They won't give you time to do your work. *Healthcare Worker, FGD2*

The only disadvantage I know is that some of them, they interfere with what you are doing, because sometimes you have to go out to take something. By the time you come, the IV fluid is running. You have your drops in place. Yes, that is the main reason, interfering with the job you are doing. *Healthcare Worker, FGD1*

Some of them, when they come, they want to tell you, do this do this to that. The birth companion. *Healthcare Worker, IDI2, age 40*

Like the IV, they say, 'it's not coming down.' They will increase it without our knowledge... They don't have the knowledge. They will just open, just rushing. You see? So it is very risky to the woman. *Healthcare Worker, FGD1*

Their patient is in pain, so they want to ease that pain. And that something that they want to do, maybe it is dangerous to the patient's life. *Healthcare Worker, FGD2*

Some... will interfere in your own work. Some will even titrate the IV. *Healthcare Worker, IDI8, age 50*

When we prepare them for section, they will give water. We tell them to starve. Maybe the patient is testing, when they are around, water. *Healthcare Worker, FGD2*

And then some patients, we tell them when they're going down for CS, don't eat anything. When you leave the patient with the birth companion, the birth companion will give them food to eat. *Healthcare Worker, FGD5*

Some will sympathize with them and give them water. They will think they are doing good to them, not mean that they are harming the patient. *Healthcare Worker, FGD2*

We tell the patient, the birth companion, that this patient should not eat anything, she is going down to the theatre for caesarean section. But they won't believe you, they will go and give the patient, some of them will give them water, some will give them rice to eat when you are not around to see them. *Healthcare Worker, FGD5*

Whenever the patient has already been booked for operation, she will starve. No water, no food. But if you leave the birth companion with the patients, maybe the patient due to her ignorance, she will request or she will ask, 'I want to drink water, I want to drink *maltina*, I want to eat rice before going down to the OT.' Then the companion will too because of ignorance she too will go and get it. And give it to the patient. So it is very risky. *Healthcare Worker, FGD2*

Native herbs

Sometimes the birth companion, when they are in the hospital, maybe you left the birth companion and the patient, went to do something. They will try to give them some native herbs, or if you give them induction, they will try to regulate the fluid. *Healthcare Worker, FGD5*

Some of them will overstep the boundary by just coming without precaution or something, your grandmother said I should give you this to drink. And most times we don't encourage it, because we give some medicines and the patient might react... Native herbs. When the attention of the nurse is not there, they will just give the woman to drink. Before you even know what is happening, just the woman, the woman like, nurse, my chest is aching as if I am feeling so much pressure in my chest, or she is itching or something. You will now be asking yourself, what have I given her that she is reacting to? Without knowing that she took something that is reacting to that medicine we give her. *Healthcare Worker, FGD1*

Some of them do give them native herbs, other tribes, they will smuggle native herbs. You know, these traditional things... you know they have this traditional belied, that when a pregnant woman is in labor, they should give her something to help her, to hasten her delivers. Some herbs. So since we know that, for now, when they come, we do educate them. And they accept. *Healthcare Worker, FGD5*

Dangerous encouragements

Even some cases, the patient will say... 'I want to use the ladies', I want to poo.' In that area, or in that situation, it's almost head at vulva – the head is pressing down. Because of the companion's ignorance, she will go and bring chamber. Tell the patient to get up and use the chamber. Which is wrong! At that time it's maybe head at vulva, the baby is coming out soon. It's very risky. So we have to monitor them. *Healthcare Worker, FGD2*

If you are talking, some will interfere. Like for example, if you are talking to the patient that you want her to lie in the left lateral position, not to lie on the back, for proper breathing. Some will say, 'nurse, no! This patient has lied too much on her side. Let her lie the way she wants to lie.' Some interfere in certain ways. Even if you tell them just for them, because the patient needs that particular position, they will try to interfere with that, you see. *Healthcare Worker, IDI8, age 50*

They will ask the patient to push before. Yeah, try to push the baby... birth companion will tell them, lie on your back, push, try and push the baby. When you are not there... Some of them will have cervical tear. Cervix is not fully dilated. *Healthcare Worker, FGD5*

Privacy

HIV and stigma

Sometimes they, the relatives, they don't keep secrets. So that's why most of the time we don't allow them to be with us... Sometimes the patient might have some conditions that we the nurses keep to ourselves. But if a relative is around, or maybe not the relative at all, maybe it's not relative to the patient. But because that person is allowed in the hospital, they saw that case, they have tendency to go out and explain to the public. So that's why we don't allow. *Healthcare Worker, FGD2*

For example, that patient is a positive case. HIV positive. And she don't want the relatives to know, or her friend to know about her condition. So at times we don't allow some inside, because we ask the patient if she needs that particular individual. Some will say, no, I don't want her to come closer to know my status, you see. Because some will talk, 'so this patient has this condition!' *Healthcare Worker, IDI8, age 50*

Let me say, the patient is positive. The patient refuses to tell the husband. Some of them don't have confidence in telling their partners about their condition... it's up to us, we the nurses, the medical staff. Because for the first time we have gotten information from the patient, we should keep it secret. *Healthcare Worker, FGD5*

The relative was able to pick up from the test that she was positive. It went viral. Because I was coming down from the labor ward, the mother-in-law or something asked me, 'is it true? Nurse, is it true that my daughter is having HIV?' I have to lie, I tell her, I don't know. I have to lie. *Healthcare Worker, FGD1*

The patient wanted to give an information to the nurse but she wasn't able to do that because she was a positive case. She was HIV-positive. She wanted to talk to the nurse, but she couldn't. She doesn't want the person to know that she's positive. *Healthcare Worker, FGD1*

She came to the facility and never told us about her status... so when she was go through delivery, the fluid spilled on my skin when I was doing delivery... So I went straight to take this kit, this kit to do this test. So I did it and she was positive. So I asked her, I wait until we finished cleaning up her, then I told the birth companion to wait outside, I want to have a private talk with the patient. So I asked her, I said, 'did you know that you are positive?' She say yes... Then I ask her, why didn't you tell me before? She said sorry, she was apologizing to me, 'I am taking my drugs, please, I am sorry. But please don't tell my birth companion.' *Healthcare Worker, FGD5*

If we want to have a private talk with the patient, we will ask them to go and take the things that we need for the baby. So during that time, at that time we will have private conversations with the patients. If we have known that the patient is positive, we will ask them. 'Have you been taking our drugs? Do you know that you are positive? Your husband know about this?' She will say no, or she will say that her husband is taking too. *Healthcare Worker, FGD5*

Companions curious about other patients

Some of them will leave their relatives, like if their relative is in room one, they will leave it and go and look at another delivery, another patient... They're very inquisitive. Some of them, they are very inquisitive. They want to know everything! *Healthcare Worker, FGD2*

They want to see everything, the want to see their own eyes. Even to the next patient... Some people are just inquisitive. So they will be disturbing, so we have to talk to them. *Healthcare Worker, FGD2*

They want to learn everything that is going on. *Healthcare Worker, IDI1, age 36*

Some will try to see exactly what is going on, but some will even go and say, 'huh? That is where the baby comes out? Inside the vagina? Huh?' *Healthcare Worker, IDI8, age 50*

Some will not pay attention to their patients, they will pay attention to the next patients, what is going on. *Healthcare Worker, FGD5*

Companions talk outside about what they witnessed

Most of these relatives, they talk a lot outside. That's why they are training us, the relative should be outside while you are doing your procedure with the patients. So now birth companionship is there. We practicing the birth companionship. While the relative is around, whenever there is extra procedure that we are doing... there will be no space for the birth companion. And that birth companion, they won't want to see the sight of that baby being delivered. When she go out now, she go talk a lot about what is happening in the labor ward. She will go and say, outside. That is another disadvantage. They should not be there when there is a special procedure on the patient. *Healthcare Worker, FGD1*

We do ask them to stand at the head for privacy. Not to go out, some will go out and talk about how the procedure was going about, so we don't want that. We just ask them to stand so we do our procedure. *Healthcare Worker, IDI8, age 50*

Some relatives, they will even try to go outside and talk... They will go and sit outside and they will explain how the delivery went about. So at times we only encourage relatives that the patient asks us to invite the labor room. Because some are very talkative. They are very inquisitive. So not all relatives we encourage inside the labor ward. *Healthcare Worker, IDI8, age 50*

I think maybe there was, because of the privacy of the patient, I think they will be thinking that they will go and say, go and expose the patient. They will go and expose all what the patient did during the giving birth. *Healthcare Worker, FGD5*

If we have birth companion by them, when there is two, three patients, some of the birth companions will go outside and say all sorts of things of the next patient, what they see. *Healthcare Worker, FGD5*

They think that the birth companion will go and tell the other people how the patient was doing, how the patient was crying, or how the patient was reacting to their pain. *Healthcare Worker, FGD5*

Some patients before do have skin disease. So you don't want the birth companion to know what is in their body. So not allowing them inside the room during the delivery. *Healthcare Worker, FGD5*

Extra work

Extra materials necessary

The relative have to change, so it's extra work for the nurses. The relative has to change before going to the theatre. Yeah so it's extra, something for the theatre, extra work, finding something... They have to have scrubs to change so that they can go in to witness. *Healthcare Worker, FGD1*

Companions faint and require attention

Some people can't withstand seeing their relatives crying... Some can withstand, some can't withstand, they'll say I won't withstand this, I will go home... It's a burden, because you have some nurses will have to leave the patients and attend to the relative. *Healthcare Worker, FGD2*

It's an additional something. You have to care for the patient, now you have to look at the birth companion also. And also some of them, when they go in, they go faint. So there is extra! *Healthcare Worker, FGD1*

Companionship and the community

Home vs. hospital delivery

That is why I say hospital is better now than at home. Because when I'm at home, I do not know anything about that one, that pregnant women should not take harmful drugs like, they have the harmful drugs pregnant women should not take... I don't know the name of the drugs, but I like the way I am know I am not supposed to take all drugs because some drugs will disturb my baby. *Companion, IDI4, age 27*

Why I choose to have birth in the hospital? Wow. Because I think that is the safest side. It's safer. Have me to have birth at home, it's not safer, because I will not have all these treatment I have here. See? Because, oh my goodness, it risks. It risks. Here is safer than home. *Delivering Woman, IDI3, age 23*

When you deliver at home it is risky, because there is no professional there to take care of you. But at the hospital, they will do anything to save you. Yes. That's why it is good to deliver in the hospital. But my first born, that time I was not having experience, that's why I deliver at home. But my second and third baby, I had the experience. As soon as my stomach ache, I went to the hospital. Because I know they will help me. *Delivering Woman, IDI11, age 32*

For me, when you give birth in a hospital, it is more better than you give birth to a midwife or your house or a TBA. Because in hospital they will have many facility that will save your life and your baby's life. So if you come to the hospital, I think that will be the better way. *Delivering Woman, FGD6*

When I was at the hospital, every morning and evening they check my pressure. No, not even morning, every shift that come. When they change shift, any nurses that come, they check your pressure. So they monitor your pressure for them to see that you are normal. But at home they will not do that. So it feels safer in the hospital to give birth at the hospital and at home. *Delivering Woman, FGD6*

Giving birth at home have so many complications. Yes. But for hospital, because even if you give birth at home, when any complication arise they will come with you to the hospital. So why should I not come to the hospital instead of risking my life and my baby? *Delivering Woman, IDI6, age 29*

To deliver in the hospital is very fine. Because when you deliver at home, they have complication at home. But when you came to the hospital, the doctors are here, the nurses are here, so all of them they will come together to see that you are deliver safe, without no problem. That's why I like to deliver in the hospital. *Delivering Woman, FGD3*

It's necessary to deliver at the hospital, because here is more advantage than giving birth at home. Because the way they treat you at the hospital, you will not have that same treatment at home. *Companion, FGD11*

I would prefer to come to the hospital to give birth because the attention and the medication there in the hospital is differ from what I should be having from home. So I think the experience of coming to deliver here is what I want to continue with. *Delivering Woman, IDI5, age 19*

Companion is learning about FBDs

She delivered. So I was so happy, with the help of god, and I mean, the technology is now improving... I was actually impressed in their work. Here I was impressed. *Companion, FGD4*

At the hospital, they help. They really help because, what they do to my sister, I saw that they do to her at the delivery room. And they were patient at her. *Companion, FGD11*

When I come with my sister, that's how I get the idea. So maybe next time, I won't give birth at home, I will come to the hospital and give birth at the hospital. Because, even at home, when I give birth, they take care of the, they take two days before they wash the baby, so. *Companion, FGD11*

I'm glad I was part of that delivery. I was very very happy, for now I understand what it means to give birth at the hospital. Because they take very care of you at the hospital. *Companion, FGD11*

I was concentrating, concentrate at the delivery room. When the doctors said, 'let the nurse person give me that other thing,' I was there watching, 'what is this name? What is they go for use? What does this thing mean?' So I ask the doctor, they say, OK, so this what this thing mean... I was asking about what they are doing. So the doctor was trying to explain for me, so it's OK, no problem. But I want to get more idea. *Companion, FGD11*

Treatment from nurses

Satisfaction with treatment

I'm so happy, I'm so glad. So I even called one of the nurse that standing by, hugging, said thank you so I was so happy. They treat us well, they make us be happy on that day. They make fun together. *Companion, IDI13, age unknown*

As for me, the one that delivered me, is like a godsend in a way. *Delivering Woman, FGD10*

They know how to talk to person, give you courage, you know? At times they make joke with you, but when you are in that mood, you will not laugh. *Delivering Woman, FGD10*

They are kind, the nurses. They are taking good care of me. *Delivering Woman, IDI11, age 32*

The reception from the nurse, I admire it, they give me good words of courage, they did embrace me. They also give me the courage and zeal for me to do it. *Delivering Woman, IDI12, age unknown*

Oh that midwife, she was really kind. She also helped to pray for me because the pain become worse now, I feel like giving more, she was there, she was helping me, yeah. She made me have courage and have the zeal to do something, to push the baby out. Talk to me, she courage me. She's my mother, I even take my number. I call her sometimes to check on her. 'Mummy, how are you?' Yeah, I made her my friend. *Delivering Woman, IDI9, age 23*

Oh I'm treated well. I'm so happy with the treatment that they give us, because the nurse make fun with us, you know they encourage us, they encourage the patients. *Companion, IDI13, age unknown*

The treatment was so nice, because they normally used to give us heated water to take their baths in the morning, they give the treatment, whenever I complain, they respond to me. So for that reason, I think that the treatment from the medical side is very good. *Delivering Woman, IDI5, age 19*

They are very caring. The nurses, they are very caring, they know how to talk to a person. *Companion, FGD11*

They do not treat us bad. They treat us good. The particular nurse that my younger sister give birth in hand, she was very nice... she is the one that call me to attention, say, come and see your younger sister's baby. So she was good, that particular one. *Companion, IDI4, age 27*

I appreciate the attitude of the nurses, their character, their response, their hospitality. We cannot be able to pay the nurses, but to pray for god to give them their blessings and give them what their heart desire with regards to the hospitality and services they have rendered to me and my daughter. *Companion, FGD4*

They are good, because they take their time, they allow me to enter, come in, enter, come in, enter, so, they treat me nicely. They treat me nicely... They are nice, they talk to me nicely, they treat me like I am one of them. *Companion, FGD11*

Treat me very nice. That day, the nurse like us. Yeah, they like us... They give me courage. *Companion, IDI10, age 26*

My mother took me to her elder sister, so I deliver there. But for now, at that time, we do not have any experience, too much experience about hospital. But now I have about many experience. When I come here, I will have medication, I will know they take good care of my baby. And also it will help me for my health, because when I go to the hospital they will say you have to take care of the, you do not have to do this, you do not have to eat this food, you do not have to take this drugs. *Companion, IDI4, age 27*

Mistreatment and abuse

Some are rude. Some things that you don't, it should not be issue, it becomes issue. *Delivering Woman, IDI6, age 29*

They are not polite to us… All the day I put myself down because I am patient, because my sister is in labor, so I put myself down, I talk to them, I beg them, so that all save me to companiate [accompany] my sister in labor room. *Companion, FGD7*

Sharing about experience with community

Companions give accurate information to community

With birth companionship, I think the name of PCMH, the bad things they are thinking about PCMH, they will change... Because some are saying that they steal, they don't allow companion or relative to be with their patient. So with that, when the companion say, no, they do a lot! We even participate... They will go out there and say PCMH has changed, has improved a lot, they

do allow us to be by our patient, to see what is going on. So I think it's very important. *Healthcare Worker, IDI8, age 50*

The patient will not give wrong information about nurses and doctors. When the birth companion is here, they will give correct information to the public. *Healthcare Worker, FGD5*

They will give information so that we have so many patients now coming to our facilities because they give correct information to them. *Healthcare Worker, FGD5*

They have trust in their companions, so they appreciate us more when we are allowing them, when we are allowing the birth companion to stay with them until they deliver, and then the birth companion go with them at home. *Healthcare Worker, FGD5*

The difference is that the companion is happy and the pregnant woman is also happy. So you go home positively. *Healthcare Worker, IDI1, age 36*

Some birth companions even met us on the streets. They shout our name, 'this is the nurse who delivered!' *Healthcare Worker, FGD5*

Encourage FBD in the community

Even when I'm at home, even my neighbor, I even told her, I said, 'please my dear, when you are in labor, come to [PCMH]. Here the nurses and the doctors are very nice. They encourage the patients, they have ask you, they even want to chat with you very nice.' I said, 'please don't sit at home and give birth, come to the hospital.' *Delivering Woman, FGD3*

[The birth companions] are the ones that will go out and tell the other people, 'that hospital, they're caring, they're loving, they look after patients.' *Healthcare Worker, FGD5*

It's also boosting our morales, that we are doing a good job... Because, they go outside and say, 'PCMH is very good, go outside and bring your patient,' so we feel proud. *Healthcare Worker, FGD5*

The companion will be happy, they will tell the community, 'when I went to give birth in the hospital, the nurses encouraged me to be near my patient, I was so happy.' Then in the community, she will encourage the community to come and be patients in the hospital. *Healthcare Worker, IDI1, age 36*
